# “In silico analysis of human TLR3 missense single nucleotide polymorphisms and their potential association with cancer”

**DOI:** 10.1038/s41598-025-05599-5

**Published:** 2025-08-22

**Authors:** Mohini Agarwal, Manish Kumar, Sarthak Dahiya, Anoop Kumar, Rupal Tripathi, Kumud Bala

**Affiliations:** 1 Therapeutics and Molecular Diagnostic Lab, J-3 Block, Amity Institute of Biotechnology, Amity University, Noida, Uttar Pradesh India; 2 Amity Institute of Pharmacy, Amity University, Noida, India; 3 National Institute of Biologicals, A-32, Sector-62, Noida, Uttar Pradesh India; 4 Rajiv Gandhi Cancer Institute and Research Centre, Delhi, India

**Keywords:** TLR-3, Cervical cancer, HPV, Missense mutation, nsSNP, In silico analysis, Biotechnology, Cancer, Computational biology and bioinformatics, Structural biology, Oncology

## Abstract

Cervical cancer is a prevalent malignancy worldwide and represents a significant health burden for women. Toll-like receptors (TLRs) are crucial components of the innate immune system and play a vital role in recognizing pathogen-associated molecular patterns. Among the TLRs, TLR3 has been implicated in the defense against viral infections, including human papillomavirus (HPV), the primary etiological agent of cervical cancer. Missense single-nucleotide polymorphisms (SNPs) in the TLR3 gene can alter its protein structure and potentially influence its function, leading to variations in immune responses and disease susceptibility. This study aimed to investigate the impact of TLR3 missense SNPs on cervical cancer development through in silico methods. A comprehensive analysis was performed using various computational tools and databases to predict the functional consequences of identified TLR3 missense SNPs. The selection criteria for SNPs included their reported association with cervical cancer or their potential effects on TLR3 structure and function. Three extremely detrimental TLR-3 nsSNPs, namely N284I (rs5743316), C37R(rs752889035), and L360P(rs768091235), have been found among the 150 nsSNPs that have so far been reported in the dbSNP database. The in silico analysis suggests that these genetic variations may contribute to the development and progression of cervical cancer by modulating TLR3 function. Further experimental studies are warranted to validate these findings and elucidate the underlying mechanisms, which may aid in developing novel therapeutic strategies for cervical cancer prevention and treatment.

**Supplementary Information:**

The online version contains supplementary material available at 10.1038/s41598-025-05599-5.

## Introduction

Cervical cancer ranks as the fourth most prevalent malignancy affecting women worldwide, arising from the epithelial tissue of the cervix^1^. Cervical cancer is one of the most successfully treated cancers when detected early. Human papillomaviruses (HPV), an extremely prevalent virus spread through sexual contact, are responsible for approximately 99% of cervical cancer occurrences^2^. The majority of occurrences of cervical cancer can be avoided with the HPV vaccine and secondary prevention techniques (screening for and treating precancerous lesions). Most HPV infections are eliminated by the immune system; however, persistent high-risk types can cause cervical cell changes that may lead to cancer^3^. It is currently unclear how the host immune system defends against HPV infection, and what the key predictors of HPV infection outcome are.

Toll-like receptors (TLRs) are pattern-recognition receptors present in the cytoplasm and cell membrane that may detect pathogen-associated molecular patterns (PAMPs). TLRs have been discovered to have a crucial role in HR-HPV-caused cervical cancer^4^. TLRs, which are required for both innate and acquired immunity, play an important part in the genesis and progression of many malignant tumors, as well as the immune system’s defense against infectious illnesses^5^. TLRs in *Homo sapiens* can be categorized into ten subclasses based on their main sequence, TLR1 through TLR10^6^. Among these TLRs, TLR1, TLR2, TLR4, TLR5, and TLR6 are present in the plasma membrane and are required for bacterial and mycobacterial population discrimination. TLR3 and TLR7, both found in endolysosomes, are essential for detecting dsRNA and ssRNA viruses, respectively^7^. TLR-8 is a gene that encodes a protein that has been associated with anogenital warts and superficial basal cell carcinoma. TLR9, which is present in the endolysosome, recognizes bacteria and viruses’ cytosine phosphate-guanine oligodeoxynucleotide (CpG-ODN). TLR expression profiles differ between TLRs. The TLR3 gene produces interferon-induced transmembrane protein 1, which is required for pathogen identification and innate defense activation. When a virus is detected, TLR3 is activated, enhancing the production of type I interferons and signaling other cells to strengthen their antiviral defenses^8,9^. Genetic polymorphisms in TLR3 have been associated with altered susceptibility to HPV infection and cervical cancer^10^.

Single-nucleotide polymorphisms (SNPs) are the most widespread form of genetic variation in humans^11^. Each SNP represents a change in a single nucleotide, the basic unit of DNA. SNPs are frequently classified into two groups based on where they are found and whether they are coding, non-coding, or intergenic. SNPs in non-coding regions may change mRNA structure, disease susceptibility, and cancer risk. Non-coding SNPs, unlike synonymous and non-synonymous substitutions in coding regions, can affect gene expression levels. By definition, synonymous replacements do not modify a protein’s amino acids but may impact its function in other ways, whereas non-synonymous substitutions are further classified as missense and nonsense variants^12,13^. A missense variant is a type of single nucleotide polymorphism (SNP) in which a single nucleotide change in the DNA sequence leads to the substitution of one amino acid for another in the resulting protein. This alteration can affect the structure and function of the protein, potentially leading to changes in its stability, activity, or interaction with other molecules. Depending on the location and nature of the amino acid change, a missense variant may have benign, deleterious, or even disease-causing effects.

SNPs in TLR genes are an important predictor of early cancer susceptibility, notably in cervical cancer. Functional non-synonymous SNPs (nsSNPs) identification requires expensive and difficult experimental approaches among the many SNPs linked to cervical cancer. As the human TLR3 gene has a huge amount of SNP data, it is critical to discover and investigate the detrimental SNPs associated with it. To prioritize the dbSNP database nsSNPs that are detrimental, the current study used a range of computational methods (dry lab work) for disease-related mutations, including PROVEAN, Mutation Assessor, PANTHER, SNAP, PhD-SNP, SNPs&GO, I-Mutant, CUPSAT, and many more on TLR3 SNPs. For the first time, the structural effects produced by the mentioned mutations, its interaction analysis & oncogenic nature of the TLR3 gene against Cervical Cancer investigation were performed.

## Materials and methods

**Fig. 1:**
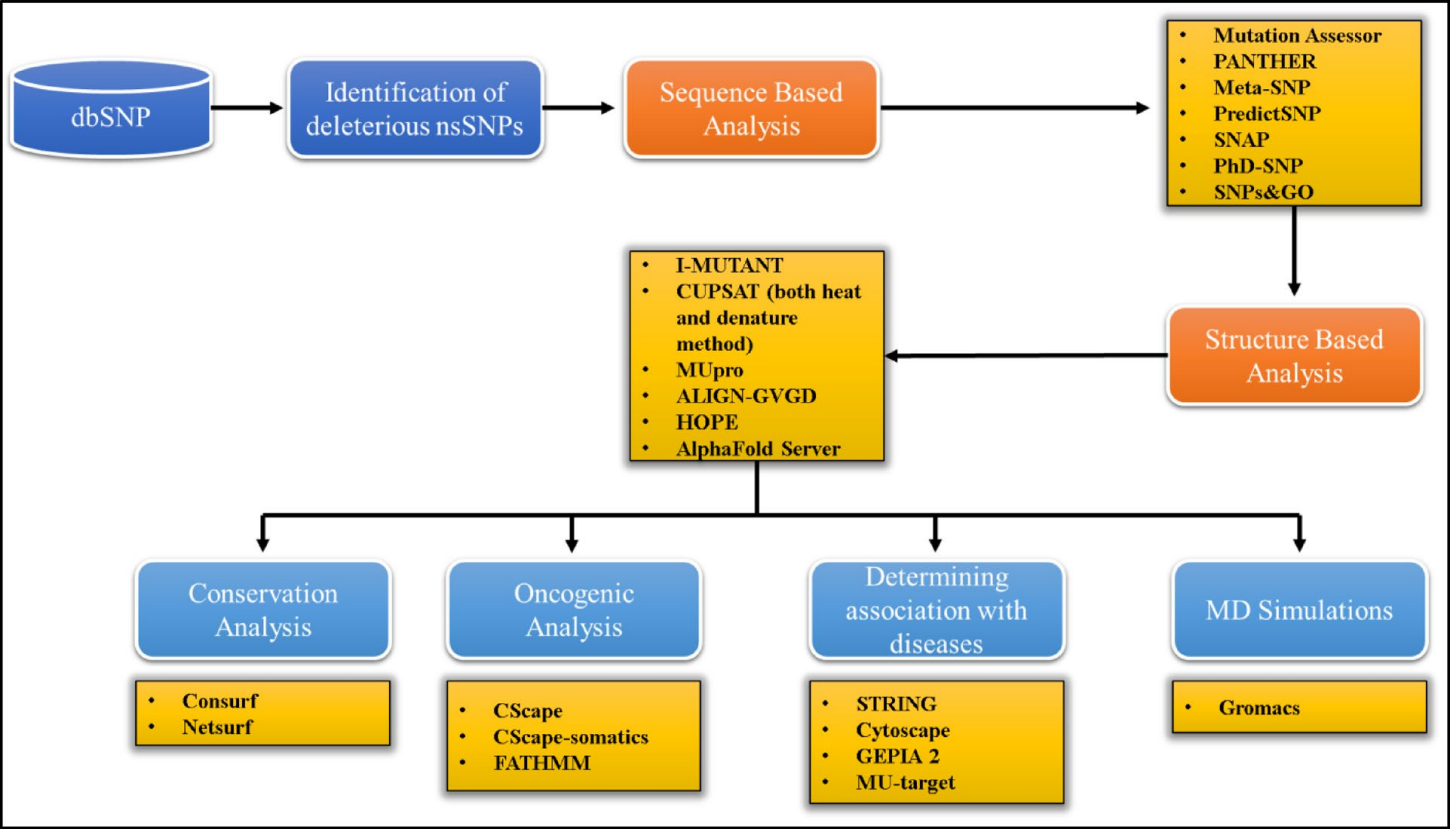
The methodology used for the analysis of the TLR3 missense SNPs.

The schematic representation of the in silico analysis performed in this study is shown in Fig. 1.

### Data collection

TLR3’s protein sequence was obtained from UniProtKB with accession ID O15455^14^. The human TLR3 gene’s nsSNPs were acquired from NCBI. The protein structure was found in the protein data bank (PDB), specifically under PDB ID: 5GS0^15^. The accession numbers of the SNPs and the direct access links to the software/tools used in the manuscript are provided in the Data availability Section (Table 1).

**Table 1:** The accession number of the SNPs and direct accessible links of the software/tools mentioned in the manuscript.

SNP IDs	Links
rs5743316	rs5743316 RefSNP Report - dbSNP - NCBI
rs199768900	rs199768900 RefSNP Report - dbSNP - NCBI
rs200214332	rs200214332 RefSNP Report - dbSNP - NCBI
rs201540925	rs201540925 RefSNP Report - dbSNP - NCBI
rs748141748	rs748141748 RefSNP Report - dbSNP - NCBI
rs752889035	rs752889035 RefSNP Report - dbSNP - NCBI
rs760275329	rs760275329 RefSNP Report - dbSNP - NCBI
rs768091235	rs768091235 RefSNP Report - dbSNP - NCBI

**Software/Tools Used**	**Links**
Meta-SNP	Meta-SNP - Meta-predictor of disease causing variants
Mutation Assessor	http://mutationassessor.org/v1
PANTHER	pantherdb.org
PredictSNP	PredictSNP: Predict SNP effect!
SNAP	SNAP (Screening for NonAcceptable Polymorphisms) | NGRL Manchester
PhD-SNAP	PhD-SNP: Predictor of human Deleterious Single Nucleotide Polymorphisms
SNPs&GO	SNPs&GO - Predicting disease associated variations using GO terms
I-Mutant	Welcome to I-Mutant2.0 Home Page
CUPSAT	CUPSAT
MUpro	Prediction of Protein Stability Changes upon Mutations
Align-GVGD	Use - Align GVGD
ConSurf	ConSurf | Evolutionary conservation profiles of proteins
NetSurfP 2.0	NetSurfP 2.0 - DTU Health Tech - Bioinformatic Services
CScape	CScape
CScape Somatic	CScape Somatic
HOPE	www3.cmbi.umcn.nl/hope/
STRING	STRING: functional protein association networks
Cytoscape	Cytoscape: An Open Source Platform for Complex Network Analysis and Visualization
GEPIA2	GEPIA 2
muTarget	| muTarget
AlphaFold	AlphaFold Server - Google DeepMind
RamPlot	Ramachandran Map
TLR3 (UniProtKB with accession ID O15455)	TLR3-Toll-like receptor 3-Homo sapiens (Human)| UniProtKB|UniProt
PDB ID- 5GS0	RCSB PDB- 5GS0: Crystal structure of the complex of TLR3 and bi-specific diabody

### Identification of deleterious nsSNPs through sequence-based tools

Different in silico or dry lab tools that could provide deleterious nsSNPs were used. The functional effect of these could be observed through these tools, and 7 of these were used. The effect of the substitution was predicted by Mutation Assessor^16^PANTHER^17^Meta-SNP^18^PredictSNP^19^SNAP^20^PhD-SNP^21^ and SNPs&GO^22^.

### Detection of damaging nsSNPs through structure-based tools

I-MUTANT^23^CUPSAT (both heat and denature method)^24^MUpro^25^and Align-GVGD^26^ tools were utilized to anticipate the impact of protein stability.

### Conservation analysis and effect of nsSNPs on protein stability and solvent accessibility of TLR3

Prediction of the conserved region for TLR3 was done by ConSurf server^27^. The ConSurf server is a bioinformatics tool for estimating the evolutionary conservation of amino/nucleic acid positions in a protein/DNA/RNA molecule based on phylogenetic relationships between homologous sequences. The evolutionarily conserved amino acid profile of the most deteriorating nsSNPs was used to gain insight into the protein’s structural and functional effect on the nsSNPs. Also, the usage of certain multiple sequences alignment tools like CLUSTAL-OMEGA and T-Coffee for TLR3 sequence of different species like *Homo sapiens*,* Mus musculus*,* Bos taurus* and *Boselaphus tragocamelus* with uniport ID O15455, Q99MB1, Q5TJ59 and Q0PV50 was used (Figure S1 & S2, Supplementary Information).

NetSurfP 2.0 program was used to determine how the most detrimental SNPs affected protein stability and solvent accessibility. Absolute surface accessibility (ASA) and relative surface accessibility (RSA) were used by the NetSurfP 2.0 web server to characterize the cumulative effects of each damaging nsSNP on the amino acids that were exposed or buried. Through the use of the NetSurfP 2.0 server, some physiological characteristics such as protein stability and solvent accessibility were taken into consideration for the potential impact of the most detrimental nsSNPs on the functional behavior of TLR3 protein^28^.

### Assessing the oncogenic nature of screened mutations and association of the damaging SNPs with cancer

For the analysis of the oncogenic nature of the screened mutations, different tools named CScape^29^ and CScape-somatic^30^ were used. Along with it, FATHMM-MKL^31^ (Predict the Functional Consequences of Non-Coding and Coding Single Nucleotide Variants) & FATHMM-XF^32^ (Enhanced Accuracy in Predicting the Functional Consequences of Non-Coding and Coding Single Nucleotide Variants) were also performed to assess the oncogenic nature of screened mutations and association of the damaging SNPs with cancer.

### Analysis of the structural assessment of the most deleterious nsSNPs of TLR3

The deleterious mutants which were filtered after structural analysis databases were used further for visualization and analysis by the HOPE (Have Your Protein Explained) server^33^. With the help of this web-based tool, how an amino acid substitution affects the protein TLR3’s physical and chemical characteristics, such as hydrophobicity, charge size differences between the wild and mutant forms, spatial structure, and protein activities can be determined.

### Protein-Protein interaction analysis & network analysis of TLR3

To get an insight on the protein-protein interaction, the STRING database which gives the TLR3 affected mutation through analysis with the interaction network and associated functions which participate in it was utilized. Any change to a protein’s structure or function can deviate the way it interacts with other molecules^34,35^.

The network was subsequently transmitted to Cytoscape (version: 3.10) for analysis. We used six methodologies from the cytoHubba plugin, including three local ranking techniques: degree, maximum neighborhood component (MNC), and maximal clique centrality (MCC), as well as three worldwide ranking algorithms: betweenness, radiality, and closeness centrality. The networks were subsequently studied using the cytoHubba plugin of Cytoscape. Nonetheless, the techniques evaluated hub proteins according to their proximity. Hub proteins were ranked according to their connectivity within the entire network, as per the global method^36^.

### Analysis of gene expression profile of TLR3 gene in association with cancer

The GEPIA2 database has been incorporated into this by performing box plot analysis of the TLR3 gene to identify the differential gene expression associated with cancer types. The box plot analysis revealed the expression of the TLR-3 gene in CESC (cervical adenocarcinoma) and UCEC (uterine corpus endometrial carcinoma)^37^.

### Determining the impact of the most detrimental nsSNPs and TLR3 gene expression on the proximate genes

The muTarget tool for examining the impact of the nsSNPs based on neighboring genes was used, and as two separate analyses “Genotype” and “Target” were present on this server, both UCEC and CESC were performed^38^.

### Three-dimensional structural assessment analysis of TLR3 protein

AlphaFold 3 server^39^ is used to perform the structural assessment of the TLR3 protein. Then Ramachandran plot was plotted using the Ramplot server.

### Molecular dynamics simulation and trajectory analysis

The three-dimensional structure of TLR3 (5GS0) was subjected to molecular dynamics simulations using the GROMACS software package^40,41^. The simulation protocol employed in this study followed previously established methods as detailed in earlier publications^42^. Protein topology parameters were derived using the CHARMM36 force field^43^. A cubic simulation box was constructed via the Gmxeditconf utility. Initially, the system underwent vacuum energy minimization using the steepest descent algorithm for 1500 steps. Solvation was achieved using the simple point-charge water model through the gmx solvate command, followed by system neutralization using gmxgenion. To eliminate steric clashes and optimize geometry, an energy minimization step was performed. This was followed by a two-phase equilibration process. The first phase involved 100 ps of NVT equilibration to stabilize the temperature at 300 K. Subsequently, 100 ps of NPT equilibration were carried out to regulate the system’s pressure and density. After successful equilibration, a 100 ns production molecular dynamics run was executed^42^.

Analysis of the simulation trajectories was performed using standard GROMACS utilities. The RMSD and RMSF for both wild-type and mutant proteins were computed using the gmxrms and gmxrmsf tools, respectively. Additionally, the radius of gyration (Rg) and solvent accessible surface area^44^ were evaluated using gmx gyrate and gmxsasa. Secondary structure elements were assessed using the do dssp module^44,45^.

### Statistical analysis

To investigate potential relationships among computationally predicted effects of high-risk nsSNPs in the TLR3 gene, a correlation analysis was conducted using a panel of bioinformatics scores, including SNAP (functional impact), I-Mutant ΔΔG (protein stability), ConSurf (evolutionary conservation), and CScape/FATHMM (oncogenic potential). Spearman’s rank correlation coefficient was calculated to assess non-parametric associations between these variables^46^. A correlation matrix was visualized using a heatmap to illustrate the strength and direction of correlations^47^. Statistical analyses were performed using Python libraries (Pandas, SciPy, Seaborn), with a significance threshold set at *p* < 0.05^47–49^.

## Results

**Fig. 2:**
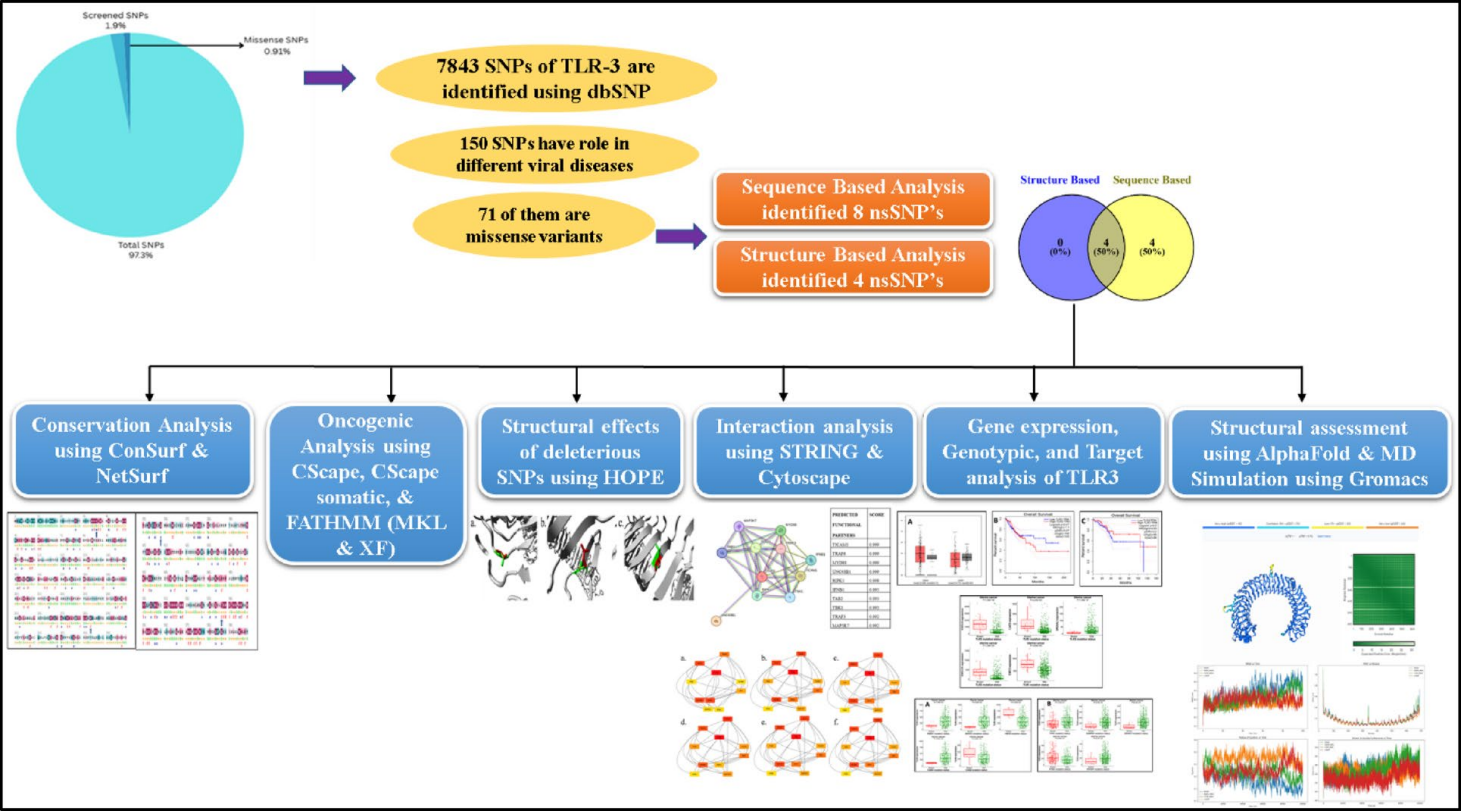
Systematic representation of the results of in silico study conducted.

The results of the study and tools that were used to find deleterious nsSNPs for the TLR 3 gene were summarized in Fig. 2.

**Fig. 3:**
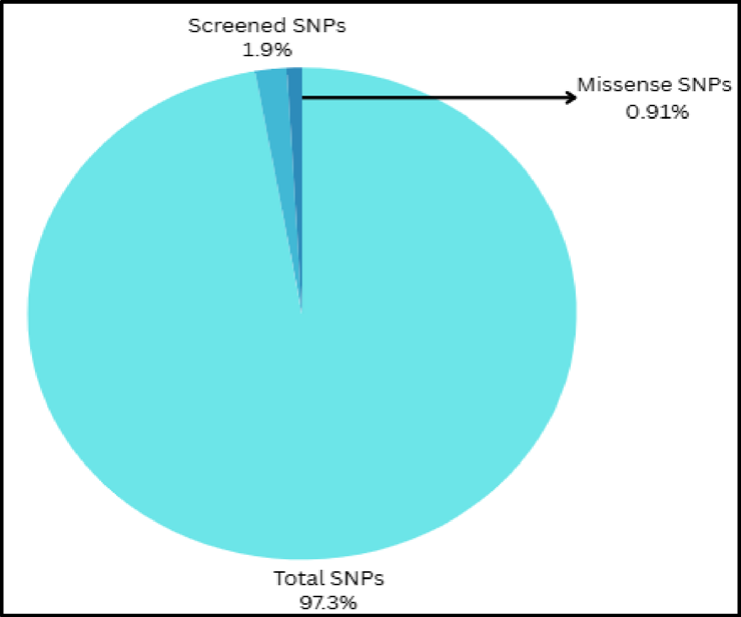
Distribution of TLR3 SNPs in dbSNP.

A total of 7843 SNPs of TLR3 gene sequence were collected from dbSNP of NCBI. Out of these, 150 SNPs were screened for selection for their deleterious role in different viral diseases. From these 150 SNPs, 71 of them were filtered to be missense variants while others were in the 3′ and 5′ UTR locations, Synonymous, non-sense, and frameshift mutations (Fig. 3).

### Screening of nsSNPs

In the current study, the TLR3 SNPs that play a damaging impact were selected. A total of 71 nsSNPs from dbSNP out of 150 were retrieved. To determine whether any of these SNPs were detrimental, they were all further assessed using various sequence-based (Table 2) and structure-based methods (Table 3).

**Table 2:** Missense nsSNPs shown to be detrimental by sequence-based methods.

SNP IDs	Mutations	Meta-SNP	Mutation Assessor	PANTHER	PredictSNP	SNAP	PhD- SNP	SNPs&GO
rs5743316	N284I	Disease	High	Probably damaging	Deleterious	Deleterious	Deleterious	Disease
rs199768900	R867Q	Disease	Medium	Probably damaging	Deleterious	Deleterious	Deleterious	Disease
rs200214332	R791G	Disease	Medium	Probably damaging	Deleterious	Deleterious	Deleterious	Disease
rs201540925	A839D	Disease	Medium	Probably damaging	Deleterious	Deleterious	Deleterious	Disease
rs748141748	A802E	Disease	Medium	Probably damaging	Deleterious	Deleterious	Deleterious	Disease
rs752889035	C37R	Disease	Medium	Probably damaging	Deleterious	Deleterious	Deleterious	Disease
rs760275329	Q538P	Disease	Medium	Probably damaging	Deleterious	Deleterious	Deleterious	Disease
rs768091235	L360P	Disease	High	Probably damaging	Deleterious	Deleterious	Deleterious	Disease

*Note: PANTHER: Between 0 and 1. (If > 0.5 mutation is predicted Disease).

PhD-SNP: Between 0 and 1.(If >0.5 mutation is predicted Disease).

SNAP: Output normalized between 0 and 1 (If >0.5 mutation is predicted Disease).

Meta-SNP: Between 0 and 1. (If >0.5 mutation is predicted Disease).

SNPs&GO : Disease probability (if >0.5 mutation is predicted Disease).

**Table 3:** Set of nsSNPs indicated by structure-based methods to be detrimental.

SNP IDs	Mutation	I-Mutant		CUPSAT (Thermal)		CUPSAT (Denature)		MUpro (Increase ↑ or Decrease ↓)				Align-GVGD
		A	B	A	B	A	B	SVM	NN	SV0M	NN	
rs5743316	N284I	↓stability	↓stability	Stabilizing	Stabilizing	Destabilizing	Destabilizing	↑	↑	↓	↓	Most likely
rs752889035	C37R	↓stability	↓stability	Destabilizing	Destabilizing	Stabilizing	Stabilizing	↑	↓	↓	↓	Most likely
rs760275329	Q538P	↓stability	↓stability	Stabilizing	Destabilizing	Destabilizing	Destabilizing	↓	↓	↓	↓	Most likely
rs768091235	L360P	↓stability	↓stability	Destabilizing	Destabilizing	Stabilizing	Stabilizing	↓	↓	↓	↓	Most likely

*Note: I-Mutant: Free energy change value (DDG) classified into largely unstable if less than − 0.5 kcal/mol, largely stable if more than 0.5 kcal/mol, and neutral if the value lies in between.

Align-GVGD: combines evolutionary conservation and biophysical properties to classify missense mutations from C0 to C65, where C65 indicates the highest likelihood of functional disruption.

MUpro: A score less than 0 means the mutation decreases the protein stability. The smaller the score, the more confident the prediction is. Conversely, a score more than 0 means the mutation increases the protein stability. The bigger the score, the more confident the prediction is.

To determine the most detrimental nsSNPs from the selected SNPs, a variety of in silico tools and algorithms, including PANTHER, Mutation Assessor, SNAP, PhD-SNP, Meta-SNP, PredictSNP, and SNPs&GO were employed. The outcomes of these tools demonstrated whether the phenotypic impacts of the amino acid alterations on protein activities were beneficial or detrimental depending on the score. A binary classifier called Meta-SNP uses the random forest-based technique to distinguish between SNPs that are linked to disease and those that are polymorphic and non-synonymous. The output of the four predictors discussed above was provided to Meta-SNP as an eight-element feature vector made up of two sets of four items each. The first group consisted of all the raw output scores from PANTHER, PhD-SNP, and SNAP variant predictions. If one of the input approaches did not generate a prediction, the method-defined default threshold for differentiating neutrals and non-neutrals as input to Meta-SNP was used. We were able to recover 23 SNPs out of 71 by this method, showing disease-causing mutations.

The mutation assessor employs a multiple sequence alignment (MSA) partitioned to represent functional specificity to identify the functional importance of a missense variant. A functional impact score was created by adding a conservation score and a specificity score. ‘Neutral’ or ‘low’ variants are expected to have no impact on protein function, whereas ‘medium’ or ‘high’ variants are expected to lead to functional modifications. By utilizing UniProt protein sequences, the mutation assessor generated its own MSA. In order to create aligned sets of families and subfamilies, it was then divided based on the boundaries of the UniProt and Pfam domains. Out of 71 mutations, we had 3 and 26 that exhibit high and medium variations, respectively.

Through the PANTHER (Protein ANalysis Through Evolutionary Relationships) Classification System, we obtained 43 mutations that were probably destructive i.e., they have a deleterious nature. The results were divided into two categories: “probably damaging” and “probably benign.” To provide a more reliable and accurate alternative to the predictions provided by individual integrated tools, the PredictSNP is a consensus classifier that combines the six best-performing prediction algorithms. PredictSNP identified 25 mutations out of 71 that were disease-causing. A total of 31 and 22 mutations, respectively were identified as being disease-causing by SNAP and PhD-SNP, two further programmes that were employed to retrieve the detrimental nature of the SNPs. SNPs&GO is an exact approach for determining whether a mutation, starting with a protein sequence, is related to a disease.

Further, we also filtered out the nsSNPs that were mostly deleterious through structural method using the PDB ID: 5GS0 through different bioinformatics tools like I-MUTANT, CUPSAT, MUpro and Align-GVGD. We employed these tools for getting a more accurate understanding of its structure and how the nsSNPs impact protein stability based on free energy change (DDG). I-MUTANT, CUPSAT, MUpro and Align-GVGD predicted 5, 3, 3 and 7 mutations according to their structure to be deleterious in nature. Among these we filtered out 4 mutations i.e., N284I, C37R, Q538P, L360P.

### Conservation analysis of TLR3

Using ConSurf, the mutations C37R and L360P had a score of 9 which showed that it had a highly conserved region and was buried inside the core region. N284I also had a score of 9 which showed a highly conserved region but was structurally exposed. Q538P had a score of 8 which was less conserved than the other three mutations and was exposed in nature.

**Fig. 4:**
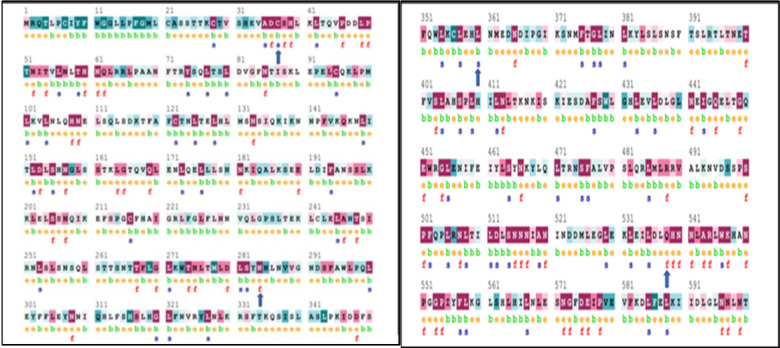
TLR3 chain A conservation analysis results predicted by the ConSurf server.

The score is analyzed on the basis of the amino acid in the variable region or conserved region. The conserved region which is scored to be 9 is color coded to be dark pink which is the most conserved. And those with a score of 1 is color coded to be blue which is the least conserved (Fig. 4). A deleterious mutation that falls under a highly conserved region is likely to be detrimental in nature.

**Table 4:** Analysis of the high-risk nsSNPs in TLR3’s evolutionary conservation profile using the NetSurfP 2.0 database.

Mutations	Class Assignment	Secondary Structure	RSA (%)	ASA (A²)
N284I	Buried	Coil	3	4
C37R	Buried	Coil	2	3
Q538P	Buried	Coil	11	20
L360P	Buried	Strand	2	4

RSA: relative surface accessibility [¼ ASA/Max ASA].

ASA: absolute surface accessibility.

NetSurfP-2.0 server has been used for predicting secondary structure, solvent, and surface accessibility. According to NetSurfP, four SNPs N284I, C37R, Q538P and L360P were buried inside the backbone of the core protein. The most detrimental nsSNPs have RSA scores ranged from 0.063 to 0.49 and ASA scores ranged from 11.55 to 105.98 (Table 4) (Figure S3, Supplementary Information).

### Oncogenic analysis of TLR3

CScape-somatic mutations isolate cancer driver mutations that arise early in tumor growth from passenger mutations that accumulate once metastasis has begun. Probability estimates, or p-scores in the range (0, 1), are used to represent predictions. Values above 0.5 are projected to be cancer drivers, while values below 0.5 are predicted to be passenger variations. Through CScape- somatic, it was seen that the mutations which had coding score as mentioned in Table 5 showed that the mutations N284I, Q538P, and L360P had values that indicated low-confidence passenger whereas C37R had a value that denoted a low-confidence driver. Cancer drivers occur fairly early in the development of the tumor. Meanwhile, passenger variants accumulate at later stages after a tumor starts to grow and usually correspond to low or no oncogenicity. However, the CScape data presents information about mutations in the chromosome 4, detailing their positions, reference bases, mutant bases, and associated coding scores. The mutation N284I, C37R and L360P were classified as oncogenic while, Q538P, showed no prediction for its coding score (Table 5).

In addition to this, FATHMM-MKL and FATHMM-XF studies were performed and all four mutations- N284I, C37R, Q538P, and L360P show significant pathogenic potential and are most likely oncogenic. Both coding and non-coding regions have incredibly high p-values (all > 0.98) according to FATHMM-MKL, indicating a high likelihood of functional disruption. In particular, there is a considerable chance that N284I (coding: 0.99256, non-coding: 0.99253) and L360P (coding: 0.99103, non-coding: 0.99325) will undergo oncogenic transformation via structural and regulatory changes. Strongly detrimental effects are shown by C37R (coding: 0.98728, non-coding: 0.99346), with its elevated non-coding score raising concerns as it may play a function in dysregulated gene expression linked to cancer (Table S1 & S2, Supplementary Information).

**Table 5:** Coding score of the filtered mutations for their oncogenic property through CScape and CScape-somatic.

CScape-Somatic					
Chromosome	Mutation	Position	Variant	Coding Score	Messages
4	N284I	187,003,691	A/T	0.120850	Passenger
4	C37R	186,997,882	T/C	0.510624	Driver
4	Q538P	187,004,453	A/C	0.116281	Passenger
4	L360P	187,003,919	T/C	0.110609	Passenger

**CScape**					
**Chromosome**	**Mutation**	**Position**	**Ref. Base**	**Mutant Base**	**Coding Score**
4	N284I	18,703,691	A	T	0.790177, oncogenic
4	C37R	186,997,882	T	C	0.624616, oncogenic
4	Q538P	18,704,453	A	C	No prediction found
4	L360P	187,003,919	T	C	0.790816, oncogenic

### Structural effects of deleterious SNPs

HOPE is designed to achieve the goal of developing a server for automatic mutant analysis that can give light on the structural implications of a mutation. The HOPE server allowed structural visualization of the three extremely detrimental nsSNPs. As well as their structural modification, amino acid characteristics, and domain, the structural information for the mutations N284I, C37R, and L360P is provided in Table 6 for all three mutations.

**Fig. 5:**
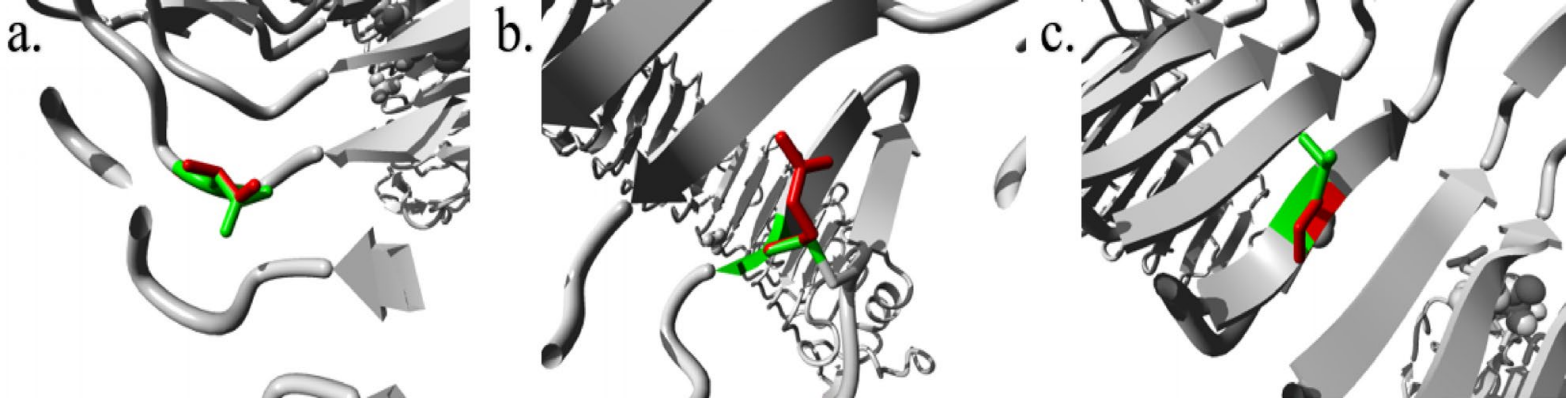
The visualization of wild-type (green) and altered (red) amino acid residues for all mutations using the HOPE Server (**a**) N284I, (**b**) C37R, (**c**) L360P.

When it comes to N284I, the altered residue is situated at a region critical to the protein’s activity and in close proximity to another region thought to be involved in binding. The mutation may interfere with how these domains interact, which could impact how signals are transmitted between them (Fig. 5a). The C37R mutation is present in a region of the protein that is crucial for its activity, and it is in close proximity to another region that is thought to be essential in binding. The mutation may interfere with how these domains interact, which could impact how signals are transmitted between them (Fig. 5b). For L360P, the altered residue is situated at a region critical to the protein’s function and in close proximity to another region thought to be essential in binding. The mutation may interfere with how these domains interact, which could impact how signals are transmitted between them (Fig. 5c).

**Table 6:** Depicts the structural impact of TLR3 mutations retrieved from the HOPE server.

N284I	C37R	L360P
		
Mutant and wild-type amino acids differ in size.	Mutants have differing charges than wild-type amino acids.	The mutant and wild-type amino acids differ in size.
Wild-type residue is less compact than mutant.	The mutant residue charges a buried residue, which may disrupt protein folding.	Compared to wild-type, mutant residues are compact.
Mutations empty the protein’s core.		
Mutant and wild-type residues differ hydrophobically.	Mutant and wild-type amino acids differ in size.	The mutation will empty the protein’s core.
Mutations weaken core hydrogen bonding, preventing folding.	Wild-type residue is smaller than mutant residue.	The altered residue is near another binding site and in a protein-critical domain.
The altered residue is near a binding site and protein-critical domain.	The protein’s core hid the wild-type residue. Due to its size, the mutant residue may not fit.	The mutation may prevent signal transmission between these sites.
These domains may interact differently due to the mutation, altering signal transmission.	Mutant and wild-type residues differ hydrophobically.	The altered residue is near other domain residues and in a protein-critical area, and this interaction may be essential for protein function.
The changed residue is near domain residues and protein-critical.	The mutation removes hydrophobic contacts from the protein’s core.	
**Common Interferences:**		
The mutation may impact this protein interaction, preventing protein function.		
The mutant residue is near a protein domain which is essential to its activity.		
The mutation may affect how these domains interact, affecting protein function.		

### Protein-Protein interaction analysis & network analysis of TLR3

**Fig. 6:**
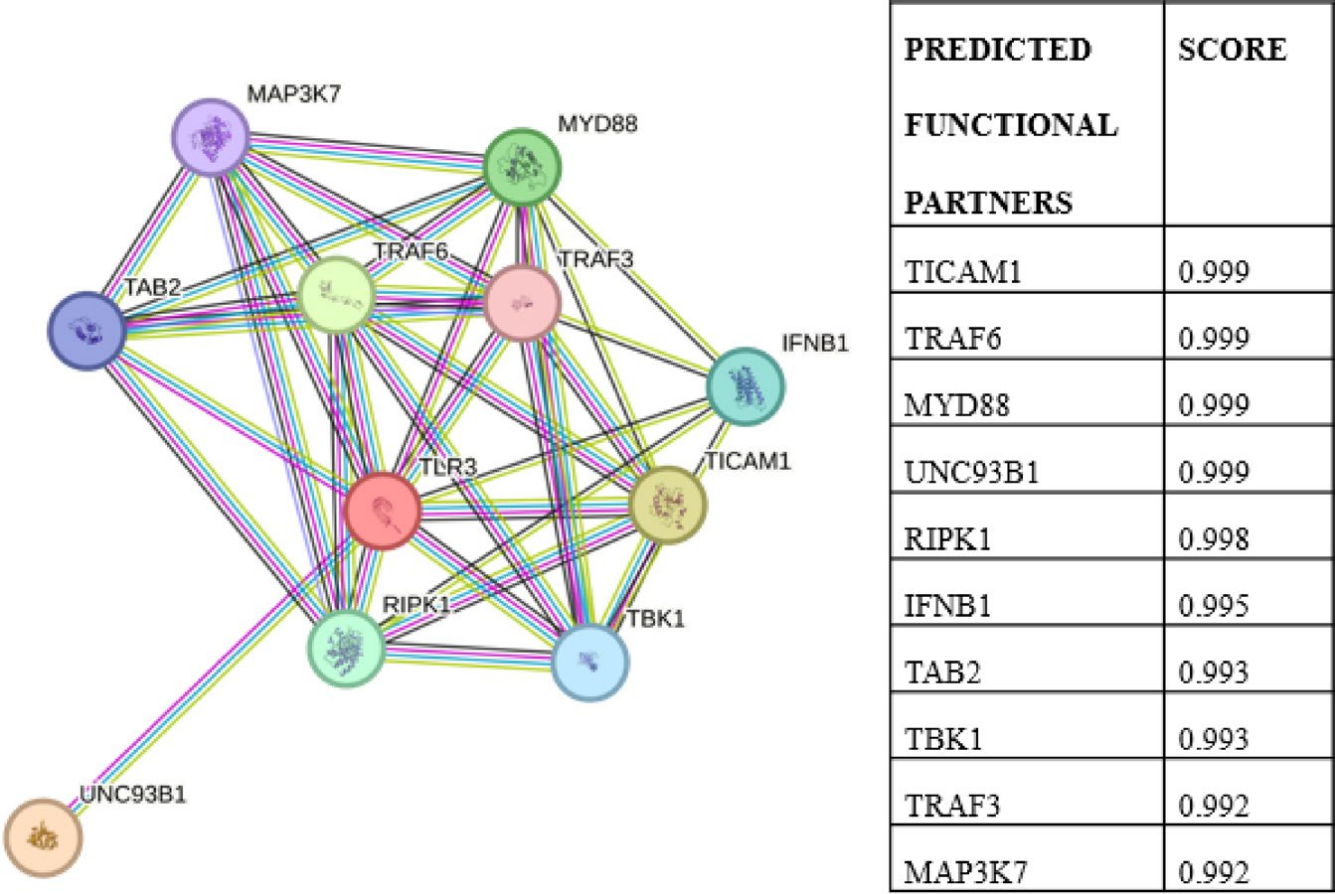
Protein-protein interaction of TLR3 gene.

**Fig. 7:**
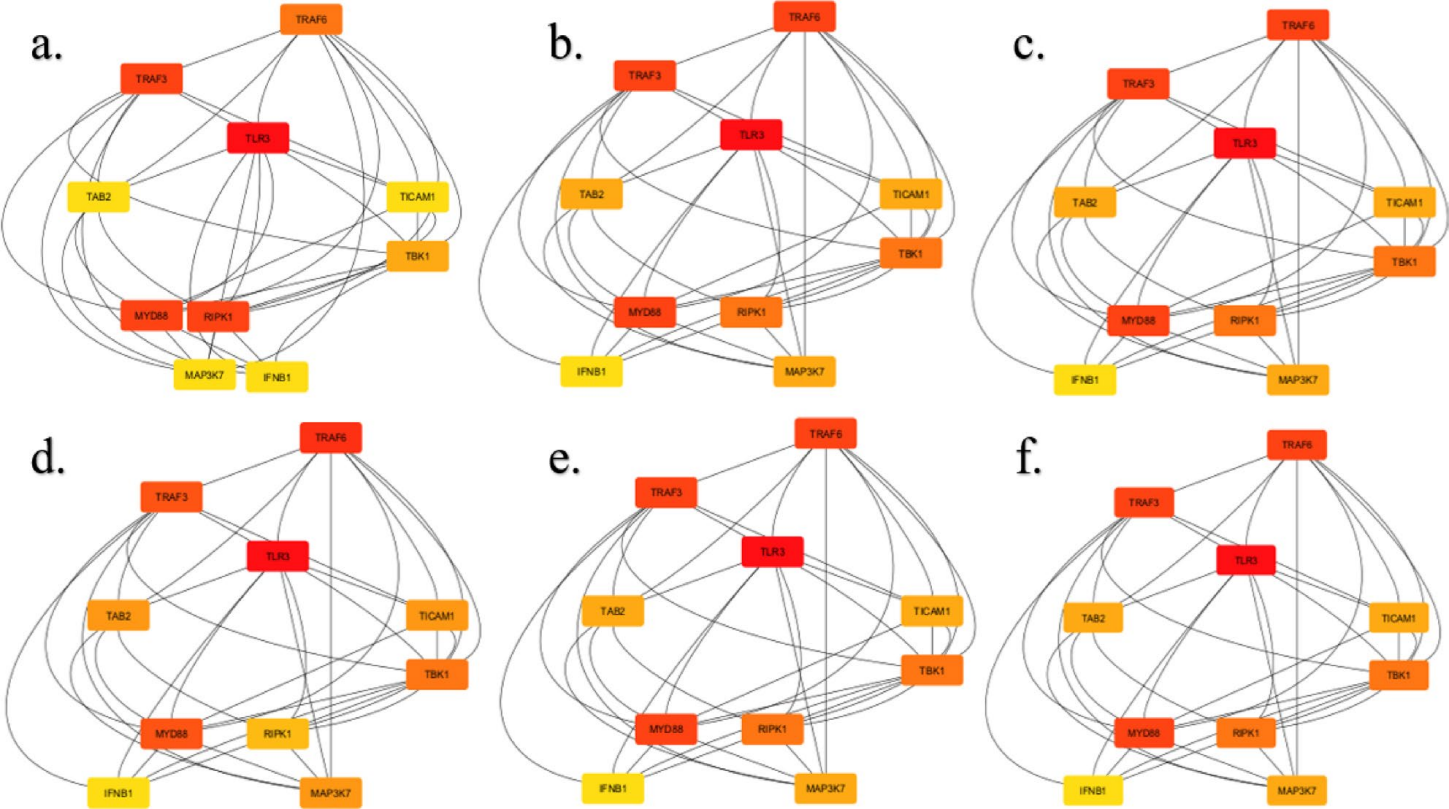
Network Analysis of genes obtained from STRING using the cytohubba plugin of Cytoscape, (**a**) Betweenness, (**b**) Closeness, (**c**) Degree, (**d**) MCC, (**e**) MNC, and (**f**) Radiality.

STRING is a database of observed and predicted protein-protein interactions. The interactions result from computational prediction, cross-species knowledge transfer, and interactions acquired from other (primary) databases; they include both direct (physical) and indirect (functional) correlations. The STRING database was used to obtain the TLR3 interaction network. ‘TLR3’ was the input name, while ‘*Homo sapiens*’ was the organism choice and those with a confidence score of 0.9 and higher were chosen. In Fig. 6, the results are displayed as nodes and edges that depict how proteins interact with one another. In Fig. 7, the network analysis reveals that TRAF6, TRAF3, and MYD88 are strongly correlated with TLR3 based on MCC, MNC, closeness, radiality, and degree, whereas apart from these three, TLR3 is also correlated with RIPK1 based on betweenness.

### Gene expression profile analysis of TLR3

**Fig. 8:**
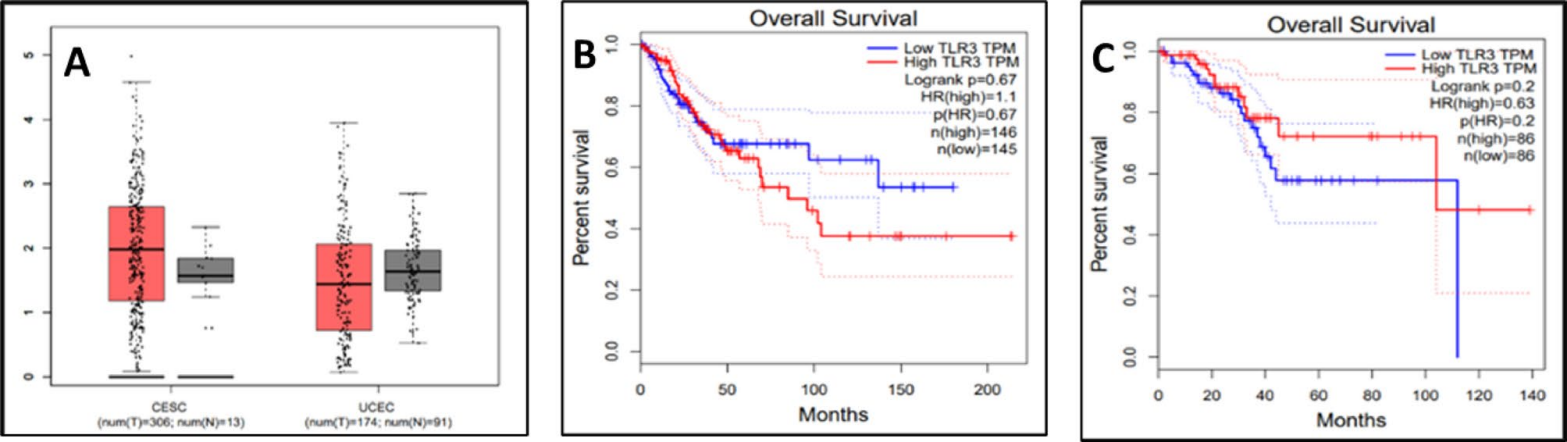
**(A)** TLR3 gene expression analysis; total % survival rate of patients for both **(B)** cervical squamous cell carcinoma (CESC) and **(C)** uterine corpus endometrial carcinoma (UCEC).

Through performing the box plot analysis using the GEPIA2 database, we can understand the differential gene expression associated with different cancer types of the TLR3 gene. Based on box plots, GEPIA2 offers differential signature score analysis. Through the results, it was observed that the two datasets of cancer types- CESC (cervical adenocarcinoma) and UCEC (Uterine corpus endometrial carcinoma) arise due to the overexpression of the TLR3 gene (Fig. 8). Generally, this means that most cancers occur due to the upregulation of the TLR3 gene.

We completed the survival analysis of the patients with CESC and UCEC using the GEPIA online database. Patients were divided into two groups, high-expression level groups and low-expression level groups, based on the median level of TLR3 gene expression. Here, the findings indicated that patients with CESC had a longer survival period in both overexpression (roughly 200 months) and underexpression of the TLR3 gene, whereas in UCEC, it was observed that patient survival periods were higher when overexpression (roughly 140) of the TL3 gene was present and lower when underexpression of the TLR3 gene was present.

### Effect of most destructive nsSNPs and expression of TLR3 gene with their neighboring genes

**Fig. 9:**
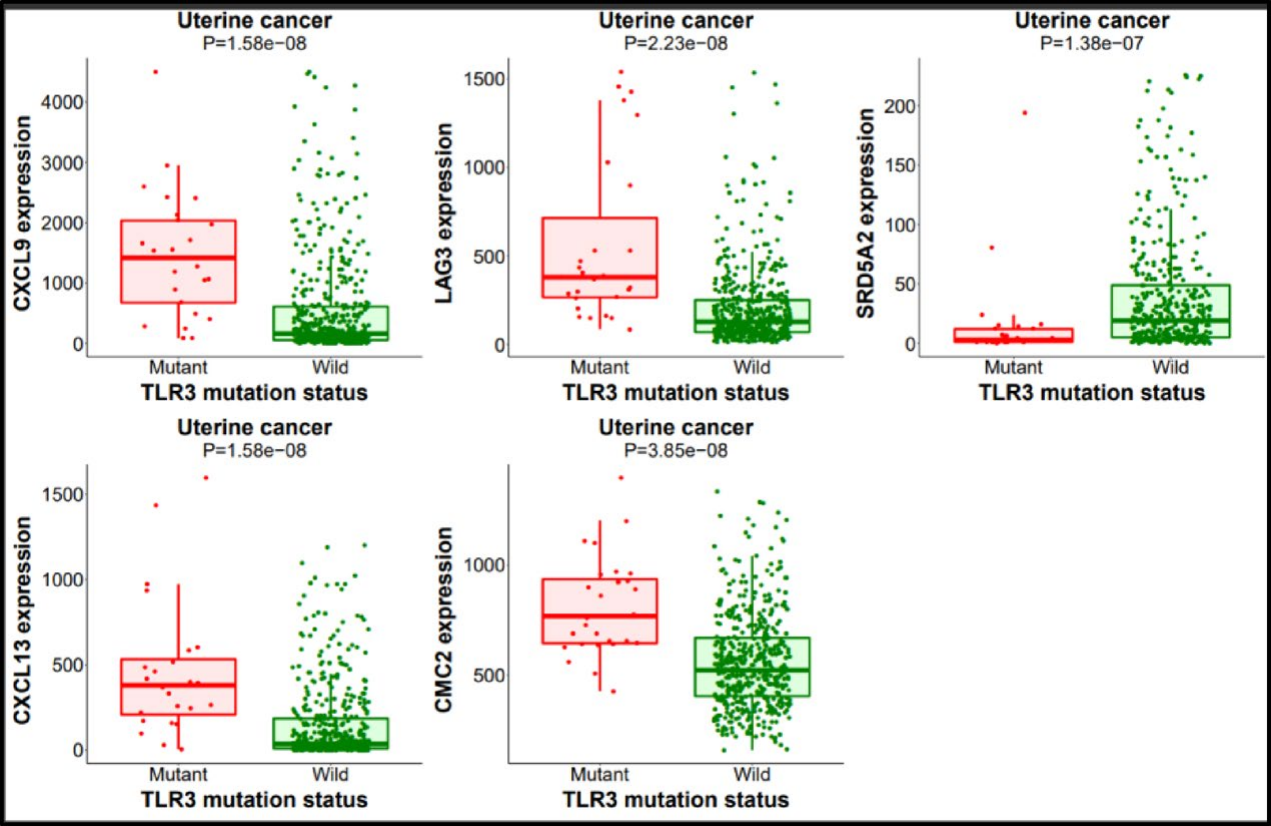
Genotype analysis of TLR3 gene.

The muTarget tool plots an expression plot and assists us in determining how the mutation affects gene expression. We used the muTarget tool for analyzing the correlation between gene expression and mutation based on the TCGA that correlates somatic mutations and gene expression in cancer. Correlations can be analyzed in two ways: The ‘Genotype’ run is for finding changes in gene expression that are related to a specific mutation and the ‘Target’ run is for finding mutations that alter the expression of target genes. The ‘Genotype’ hypothesis findings for uterine cancer showed that the expression of the genes CMC2, CXCL13, CXCL19, LAG3, SRD5A2, and others had changed (Fig. 9).

**Fig. 10:**
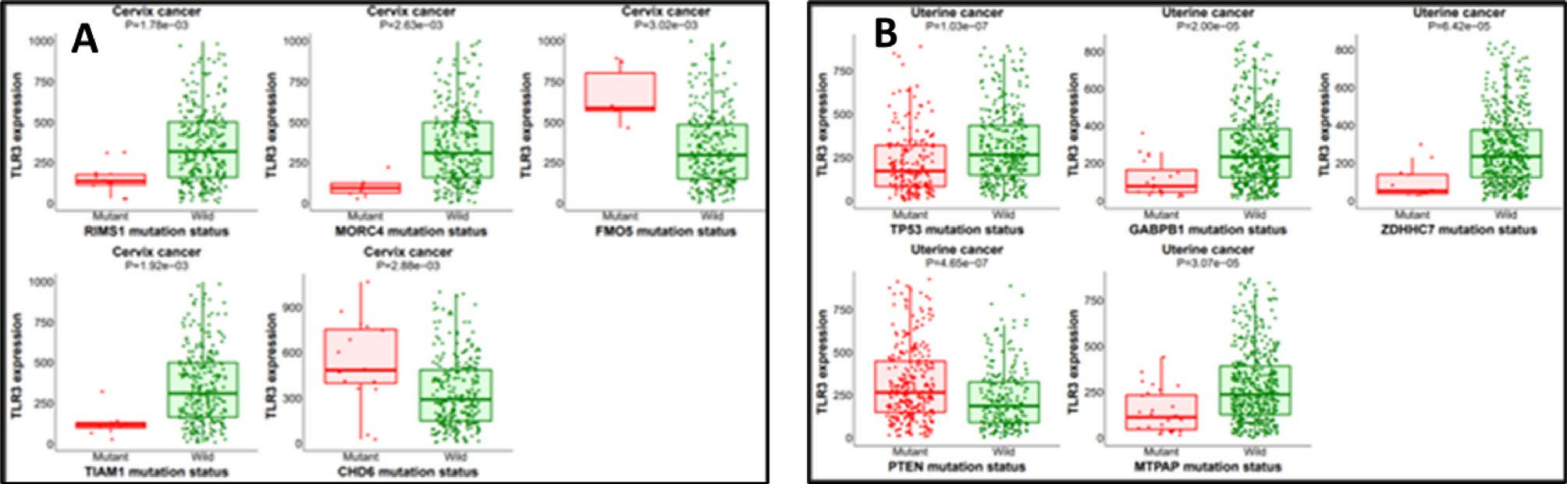
Target analysis of TLR3 gene **(A)** Cervical cancer and **(B)** Uterine cancer.

The number of patients was too low (less than 10) to make any conclusions, and TLR3 was unable to change the expression of the nearby gene, hence there were no results for cervical cancer presented in the database. The TLR3 gene was used as input for both uterine and cervical cancer in the following Target run. According to our findings, the CHD6 gene exhibited higher expression than the RIMS1, MORC4, FMO5, TIAM1, and other genes in cervical cancer (Fig. 10A). Alternatively, for uterine cancer, the expression can be influenced by TP53, GABPB1, ZDHHC7, PTEN, MTPAP and other genes. When compared to its counterparts, PTEN in this category exhibited the greatest change in uterine cancer gene expression (Fig. 10B). Mutations in any interacting gene partners in the gene-gene network affect their neighboring genes, which may result in cancer formation, according to the muTarget study findings.

### Three-dimensional structure assessment of TLR3

**Fig. 11:**
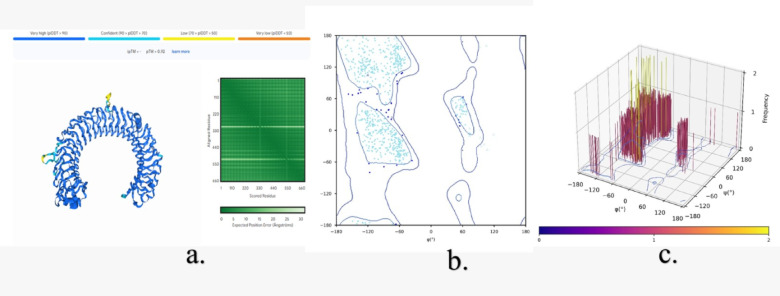
**(a)** Structural assessment of TLR3 without mutations, **(b)** 2-D Ramachandran plot analysis (green, blue, and red (dots/triangles) represent torsion angles of favored, allowed, and disallowed regions respectively; dot represents residues other than glycine and triangles represents glycine), and **(c)** 3-D Ramachandran Plot (bar represents the frequency of torsion angles).

**Fig. 12:**
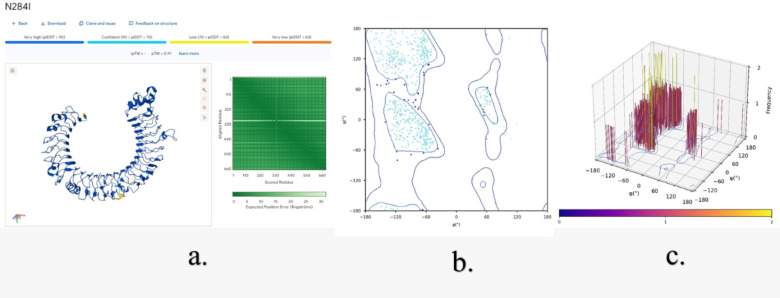
**(a)** Structural assessment of TLR3 with N284I mutation, **(b)** 2-D Ramachandran plot analysis (green, blue, and red (dots/triangles) represent torsion angles of favored, allowed and disallowed regions respectively; dot represents residues other than glycine and triangles represents glycine), and **(c)** 3-D Ramachandran Plot (bar represents the frequency of torsion angles).

**Fig. 13:**
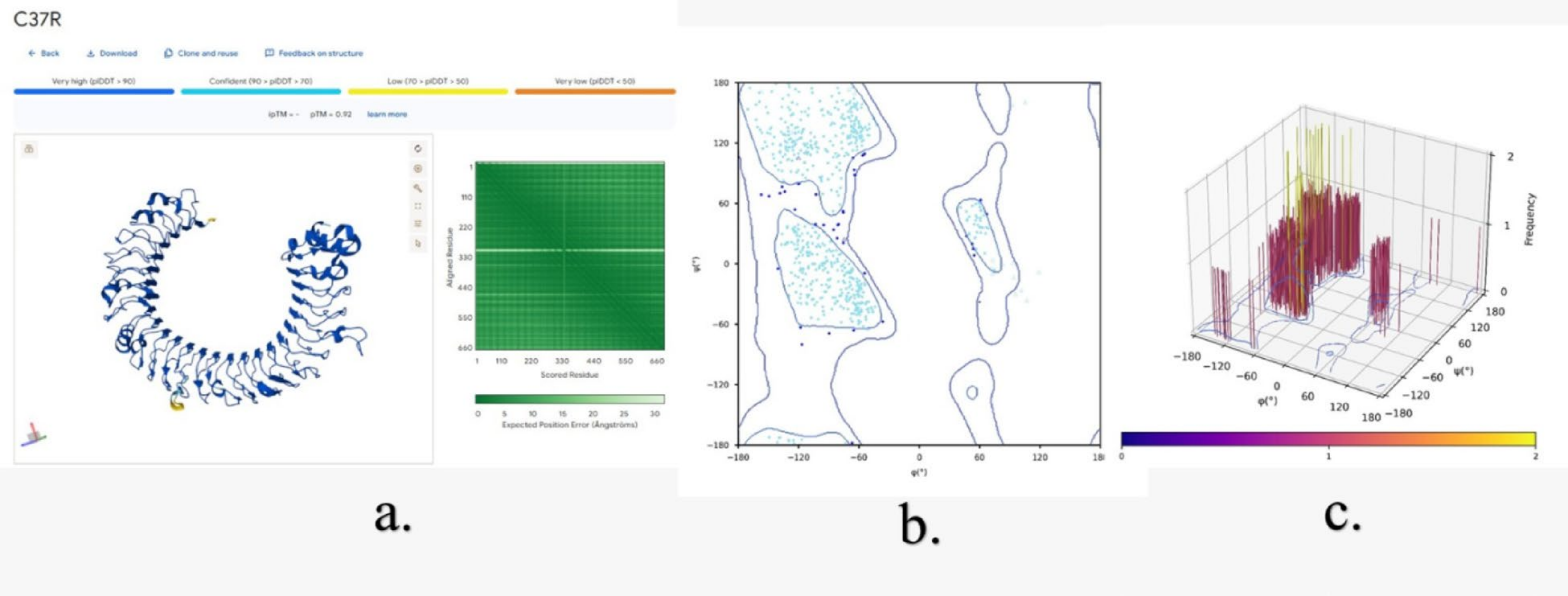
**(a)** Structural assessment of TLR3 with C37R mutation, **(b)** 2-D Ramachandran plot analysis (green, blue, and red (dots/triangles) represent torsion angles of favored, allowed and disallowed regions respectively; dot represents residues other than glycine and triangles represents glycine), and **(c)** 3-D Ramachandran Plot (bar represents the frequency of torsion angles).

**Fig. 14:**
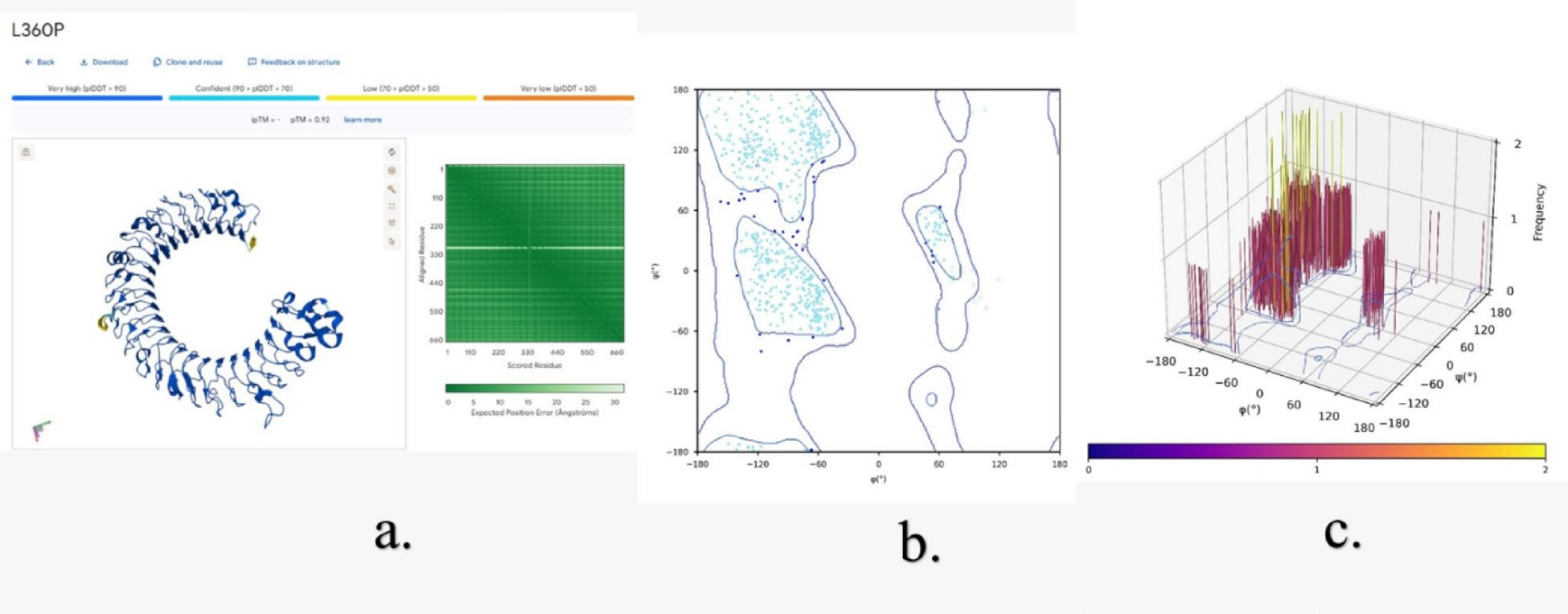
**(a**) Structural assessment of TLR3 with L360P mutation, **(b)** 2-D Ramachandran plot analysis (green, blue, and red (dots/triangles) represent torsion angles of favored, allowed and disallowed regions respectively; dot represents residues other than glycine and triangles represents glycine), and **(c**) 3-D Ramachandran Plot (bar represents the frequency of torsion angles).

The Three-dimensional structure assessment of TLR3 without mutations (Fig. 11) and with mutations (N284I, C37R and L360P) (Figs. 12 and 13, &14) is performed with the help of the Alphafold 3 server. The projected template modeling (pTM) score and the interface predicted template modeling (ipTM) score are both generated from a measure known as the template modeling (TM) score. This assesses the precision of the complete structure. A pTM score exceeding 0.5 indicates that the predicted fold of the complex may closely resemble the actual structure. The ipTM evaluates the precision of the anticipated relative placements of the subunits inside the complex. Values beyond 0.8 indicate robust, high-quality predictions, whilst values below 0.6 imply a probable failure in prediction. ipTM values ranging from 0.6 to 0.8 represent an ambiguous zone in which forecasts may be accurate or inaccurate. The 2D & 3D Ramachandran plots were plotted for both structures without (Fig. 11b and c) and with mutations (Figs. 12b and c, 13b and c and 14b and c). In Fig. 11a, a structural assessment of TLR3 without mutations gave a pTM score of 0.92, while Figs. 12a, 13a and 14a respectively showed structural assessment of TLR3 with mutation N284I gave a pTM score of 0.91, TLR-3 with mutation C37R gave a pTM score of 0.92, and TLR3 with mutation L360P gave a pTM score of 0.92, all scores indicating robust and high-quality structural predictions. (Figure S4, S5, S6 & S7 Supplementary Information).

### Molecular dynamics simulation and trajectory analysis

**Fig. 15:**
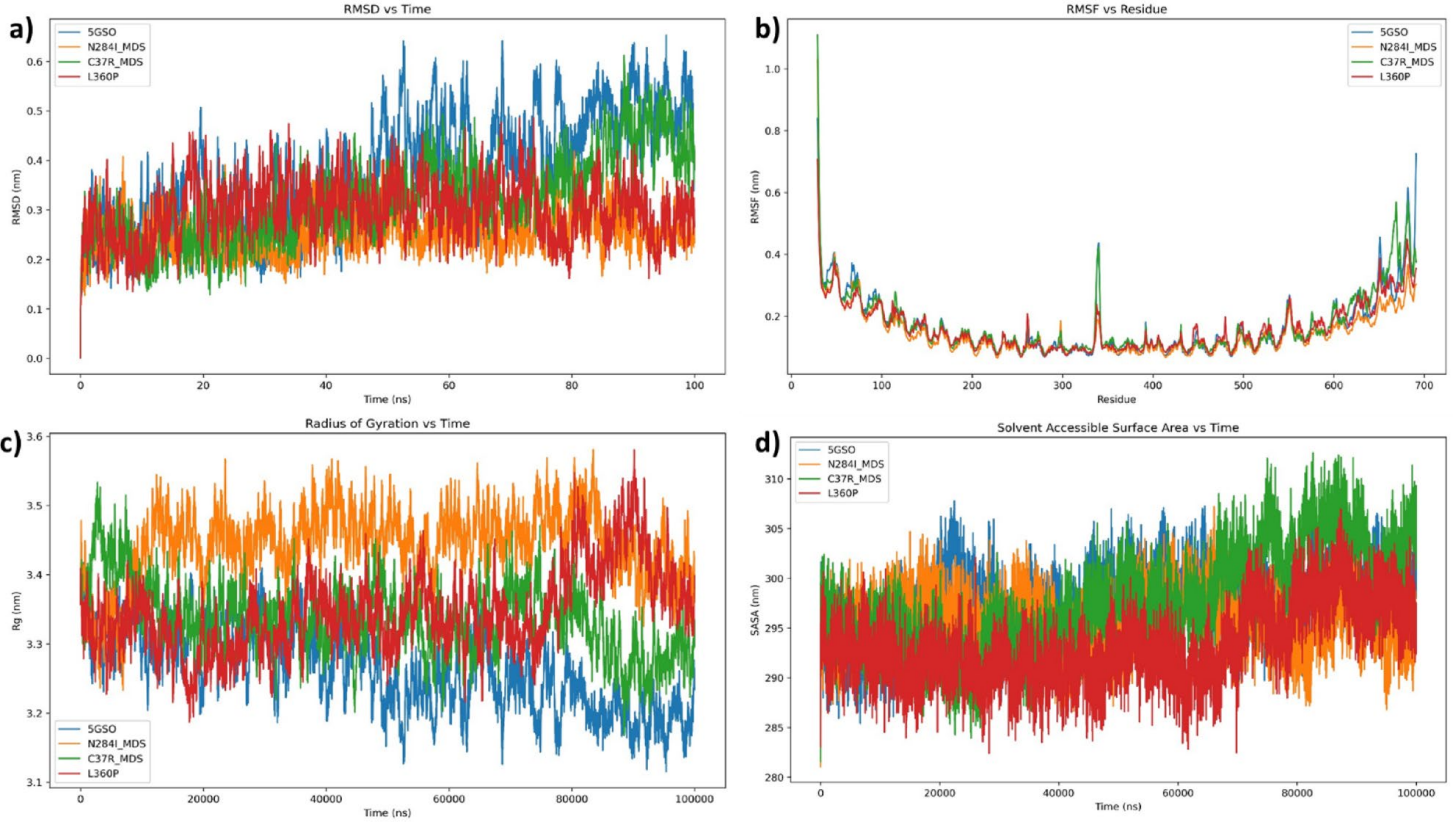
Protein & its mutation analysis of all four plots—(**a**) RMSD, (**b**) RMSF, (**c**) Radius of gyration, and (**d**) SASA for 100 ns or 100,000 ps [5GS0 (blue), N284I (orange), C37R (green), and L360P (red)].

The Root Mean Square Deviation (RMSD) plot provides an overview of the structural stability of the protein throughout the simulation period. The wild-type 5GS0 (blue) shows bigger and more stable variable RMSD values than the mutants in the RMSD vs. Time plot (Fig. 15a), suggesting greater structural deviation and more extensive stability during the simulation. In contrast, the N284I mutant (orange) exhibits the lowest and most reliable RMSD, indicating a noticeably more stable conformation. The C37R (green) and L360P (red) mutants are in the between, with moderate RMSD variations. According to this pattern, the wild-type structure is more vulnerable to dynamic shifts under the simulated conditions, whereas the N284I mutation may give increased conformational stiffness or structural integrity.

The Root Mean Square Fluctuation (RMSF) plot reveals localized flexibility within the protein structure by displaying the time-averaged fluctuation of each residue. The RMSF versus Residue plot (Fig. 15b) demonstrates per-residue flexibility. The general profile of all variants is similar, with higher fluctuation in the N- and C-terminal regions-which is to be expected given that they are generally disordered. N284I and C37R show somewhat greater fluctuations in the core region (about residues 300–400), despite the fact that the RMSF trends are generally conserved across all proteins. These results suggest localised instability or conformational flexibility in areas that may be important in functional interactions.

The Radius of Gyration (Fig. 15c), which measures structural compactness, shows that the wild-type 5GS0 exhibits greater fluctuations and tighter packing, indicating sporadic transitions between compact and slightly relaxed states. With continually higher Rg values, N284I stands out once more, suggesting a more expansive structure overall. Compared to 5GS0, C37R and L360P similarly show higher Rg values, albeit they fluctuate more than N284I. This implies that the mutants, especially N284I, retain a stable but less compact shape, which may affect how they function biologically.

The Solvent Accessible Surface Area (SASA) plot provides insights into how much of the protein’s surface is accessible to the solvent, reflecting changes in protein folding and hydrophobic core exposure. The Solvent Accessible Surface Area (SASA) plot (Fig. 15d) showed 5GS0 and C37R (blue and green) comparatively greater surface exposure than L360P and N284I. It’s interesting to note that N284I exhibits lower SASA despite being compact in Rg; this could be a sign of stronger surface contacts or hidden residues that restrict solvent access. L360P exhibits a comparable pattern.

### Statistical analysis

To evaluate the relationships between various computational prediction scores associated with high-risk nsSNPs in the TLR3 gene, we conducted a Spearman correlation analysis. The results are visualized in the correlation heatmap (Figure S10, Supplementary Information). A strong positive correlation was observed between the SNAP score and the CScape score (*r* = 0.96), suggesting that mutations predicted to be functionally damaging by SNAP are also likely to be predicted as oncogenic by CScape. Similarly, the I-Mutant ΔΔG values showed a strong inverse correlation with both SNAP (*r* = −0.87) and CScape scores (*r* = −0.97), indicating that SNPs predicted to decrease protein stability are also associated with higher functional and oncogenic impact. ConSurf scores, which reflect evolutionary conservation, showed weak correlations with other metrics (SNAP: *r* = 0.17, I-Mutant: *r* = −0.10, CScape: *r* = −0.10), suggesting that conservation alone may not be sufficient to predict functional or oncogenic significance in this context.

## Discussion

In this current study, we have shown the role of TLR3 associated with cervical cancer by computational approach. After running a set of databases, we found that among 71 missense mutations, 3 of them were highly detrimental in nature. These retrieved mutations namely N284I, C37R and L360P were further involved with different databases like HOPE server, STRING, GEPIA2 and muTARGET tool for gaining more understanding.

Previous studies have demonstrated that TLR3 is aberrantly expressed in several cancers, including cervical cancer. For instance, Hasimu et al., (2011)^50^ reported elevated TLR3 expression in cervical intraepithelial neoplasia and cervical squamous cell carcinoma tissues compared to normal cervical tissues, suggesting a role in cervical carcinogenesis. Similarly, studies have shown that TLR3 expression correlates with apoptosis, proliferation, and angiogenesis in hepatocellular carcinoma, and serves as a prognostic biomarker in renal clear cell carcinoma. These findings underscore the significance of TLR3 in tumor biology across various cancer types^51^.

Firstly, after retrieving the missense SNPs from NCBI, the mutations were incorporated into certain sequence-based tools like Mutation Assessor, PANTHER, Meta-SNP, PredictSNP, SNAP, PhD-SNP and SNPs&GO. These databases helped us to filter out the most deleterious mutations. To get more precise results of damaging mutations, we considered structural-based methods as well like the I-MUTANT, CUPSAT, MUpro and Align-GVGD. Through this, we sorted out 4 mutations that were highly detrimental in nature and these are N284I, C37R, Q538P and L360P. To understand the oncologic nature of these mutations, we used the databases like CScape, CScape-somatic and FATHMM, which showed that N284I, C37R and L360P were the more damaging mutations that could be interrelated to cancer. N284I has proven to be highly detrimental to different diseases other than cancer. Because of its association with impaired TLR3 signaling in vitro, it’s been said that N284I is highly related to viral infections in humans^52^. Previous studies have already implicated N284I in impaired TLR3 signaling and increased susceptibility to viral infections^53^suggesting its pathogenic role beyond cancer.

In addition to this, C37R of TLR3 mutations has been predicted for the first time through computational analysis (dry lab) to be highly deleterious and potentially associated with cervical cancer. The mutation C37R, although not previously reported in a cancer context to our knowledge, was shown in our study to significantly impact protein structure and stability, and is proposed here for the first time as a novel cancer-related TLR3 mutation. However, further experimental validation is needed to confirm its impact on cancer and other diseases.

Another mutation namely L360P, has been proven to be oncogenic. To support this, previous studies has shown that L360P is an HSE-causing (herpes simplex encephalitis) TLR3 mutation among the others that are P554S, E746X, G743D + R811I, and R867Q^54^. It has also been reported that heterozygosity for the HSE-causing L360P or G743D + R811I allele causes AD TLR3 deficiency in fibroblasts due to negative dominance and haploinsufficiency, respectively^54^. Therefore, our study expands on these findings by suggesting that these N284I, C37R and L360P may also contribute to oncogenic processes in cervical cancer, thereby connecting TLR3 immune dysfunction to tumor biology.

To explore the functional implications of the identified high-impact nsSNPs (N284I, C37R, Q538P, L360P), we extended our analysis to include protein interaction networks, gene expression profiles, and mutation-expression correlations. STRING-based network analysis revealed strong interactions between TLR3 and key immune signaling proteins such as TRAF3, TRAF6, and MYD88, suggesting that these mutations could affect downstream immune pathways consistent with earlier findings that describe their involvement in TLR3-mediated NF-κB signaling^55^.

Gene expression profiling using GEPIA2 revealed that TLR3 expression is upregulated in both cervical squamous cell carcinoma (CESC) and uterine corpus endometrial carcinoma (UCEC). Interestingly, survival analysis showed improved outcomes in UCEC patients with high TLR3 expression, whereas results for CESC were inconclusive, reflecting the context-dependent dual role of TLRs in tumor promotion and suppression. Furthermore, the muTarget analysis provided insight into mutation-associated expression shifts in neighboring genes. CHD6 showed the highest expression in cervical cancer, while TP53 and PTEN exhibited the greatest expression changes in uterine cancer. These findings point to potential network-level interactions, in which TLR3 mutations may influence the regulation of cancer-critical genes indirectly, in line with systems biology studies.

To support the structural analysis, AlphaFold was used to generate separate structural models for each point mutation-C37R, N248I, and L360P-rather than combining all mutations into a single structure. This approach was adopted to better understand the individual structural consequences of each mutation without the confounding effects of multiple simultaneous substitutions. By modeling each mutant independently, we were able to observe localized conformational changes specific to each variant, allowing for a clearer interpretation of their potential impact on protein stability and function. This refinement improves the accuracy of structural comparison with the wild-type (PDB ID: 5GS0) and enhances the clarity of our structural analysis. Additionally, Sankari et al., (2024)^56^ and Kamal et al., (2025)^57^ also utilized Alphafold to generate accurate 3D models, enabling detailed visualization and functional analysis of mutant proteins. Our findings are in line with the study by Jumper et al., (2021)^58^which validates AlphaFold’s accuracy for modeling high-resolution protein structures and guiding mutational impact studies.

In our study, the molecular dynamics simulations revealed notable structural differences between the TLR3 wild-type (5GS0) and its mutants (N284I, C37R, and L360P), as assessed through solvent accessible surface area (SASA), radius of gyration (Rg), RMSD, and RMSF. Among these, N284I exhibited the lowest RMSD and the most stable conformation, indicating enhanced structural integrity. This stability was further supported by RMSF analysis, which showed preserved flexibility across the mutants, though some increased fluctuations were noted around residues 300–400 in N284I and C37R. Furthermore, Rg analysis demonstrated that N284I maintained a more stable but expanded structure, potentially facilitating better ligand accommodation. SASA data revealed that N284I and L360P had reduced solvent exposure, suggesting tighter internal packing, a characteristic often linked to structural stabilisation in similar systems. In recent studies, molecular dynamics (MD) simulations have been employed to explore the structural effects of point mutations in TLR3 and TLR4. Mahita et al., (2018)^59,60^ investigated the TLR3 wild-type (WT) homodimer and TLR3 A795P homodimer to understand the impact of key mutations on the protein’s structural behavior. Their study highlighted subtle conformational changes induced by phosphorylation and point mutations in the TIR domains of TLR3, providing deeper insights into the molecular alterations that occur in these regions. Similarly, Prakasam et al., (2023)^61^ used MD simulations to examine structural changes in TLR4 induced by point mutations, further advancing our understanding of how such mutations influence TLR proteins and their functions.

In addition to this, correlation analysis further reinforced the findings. A strong positive correlation between SNAP and CScape scores (*r* = 0.96) indicated that mutations predicted to be functionally damaging are also likely to be oncogenic. Additionally, I-Mutant ΔΔG scores were inversely correlated with both SNAP (*r* = −0.87) and CScape (*r* = −0.97) values, highlighting the role of structural destabilization in disease progression. In contrast, ConSurf scores had weak correlations with other metrics, suggesting that while conservation indicates evolutionary importance, it does not alone predict pathogenicity.

However, this study concentrates on the most detrimental nsSNPs linked to cervical cancer; yet the impact of these SNPs may also be examined in relation to other serious disorders. Despite the estimations of this study being derived from several well-known tools and algorithms, comprehensive experimental research and large-scale population studies, in conjunction with clinical trials, are requisite prior to the use of the principal findings for clinical reasons. Moreover, these computational predictions are currently being validated through ongoing wet-lab experiments in our laboratory, which will provide experimental support for the in silico findings related to TLR3 SNPs.

## Conclusion

This study has predicted that TLR3 is related to cervical cancer with the help of the missense SNPs retrieved and the different in silico techniques involved. The TLR3 gene has undergone analysis of its nsSNPs since it has been linked to a number of complex illnesses. Four extremely damaging TLR3 nsSNPs namely N284I(rs5743316), C37R(rs752889035), Q538P(rs760275329), and L360P(rs768091235) have been found out of the 150 nsSNPs that have so far been reported in the dbSNP database. Based on a variety of evaluations, N284I, C37R and L360P were identified as the three most detrimental mutations. Although in silico methods cannot completely replace physical and frequently conclusive testing processes and methodologies, the current work is regarded to be useful for future research efforts that target TLR3 to treat cervical cancer.

## Electronic supplementary material

Below is the link to the electronic supplementary material.

Supplementary Material 1

## Data Availability

Data is provided within the manuscript.
