# Supplementary material for: “In silico analysis of human TLR3 missense single nucleotide polymorphisms and their potential association with cancer”
Source: Sci Rep. 2025 Aug 22;15:30837. doi: 10.1038/s41598-025-05599-5 (PMC12373945; doi:10.1038/s41598-025-05599-5)
Supplement: Supplementary file 1 — Supplementary Material 1 [file 41598_2025_5599_MOESM1_ESM.docx]

Supplementary Information

“In silico analysis of human TLR3 missense single nucleotide polymorphisms and their potential association with cancer”

Mohini Agarwal^1^, Manish Kumar^1^, Sarthak Dahiya^2^, Anoop Kumar^3^, Rupal Tripathi^4^ & Kumud Bala^1^*

Affiliation of the Authors

1. Amity Institute of Biotechnology, Amity University Uttar Pradesh, Noida, India
2. Amity Institute of Pharmacy, Amity University, Noida, India
3. National Institute of Biologicals, Sector-62, Noida, India
4. Rajiv Gandhi Cancer Institute and Research Centre, Delhi, India

*Corresponding Author- Prof. (Dr.) Kumud Bala, Professor

Therapeutics and Molecular Diagnostics Lab, Lab No.-319, J-3 Block, Amity Institute of Biotechnology, Amity University, Noida, Uttar Pradesh

[kbala@amity.edu](mailto:kbala@amity.edu), 9811292212.

T-COFFEE RESULTS


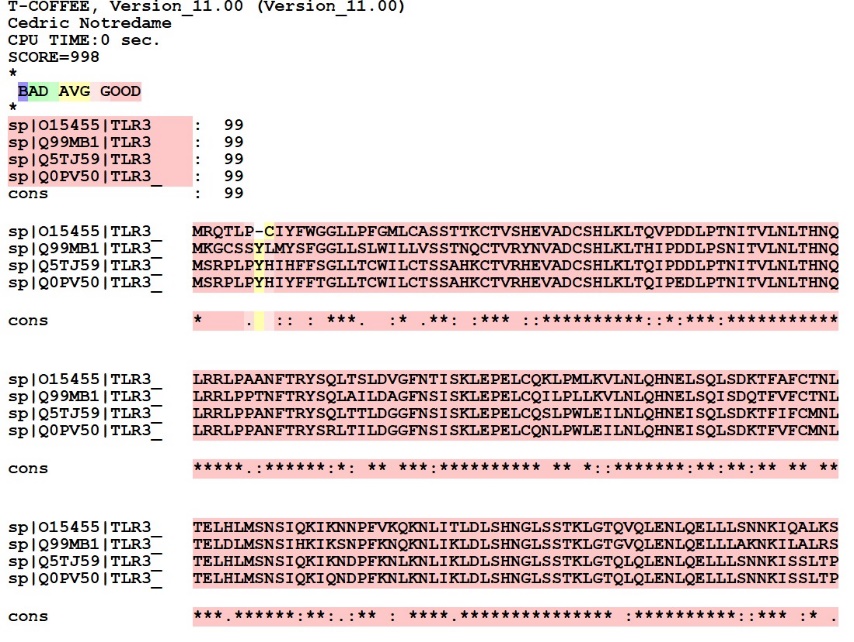


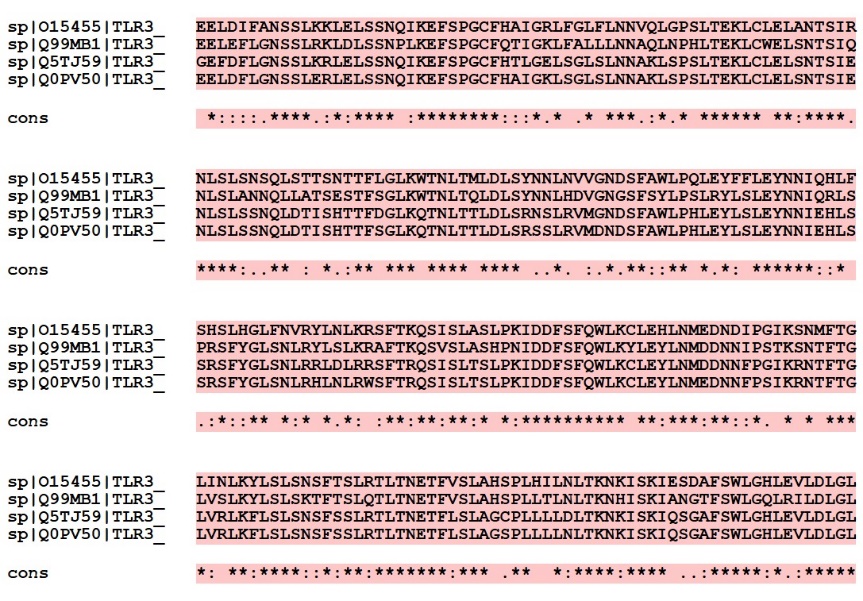

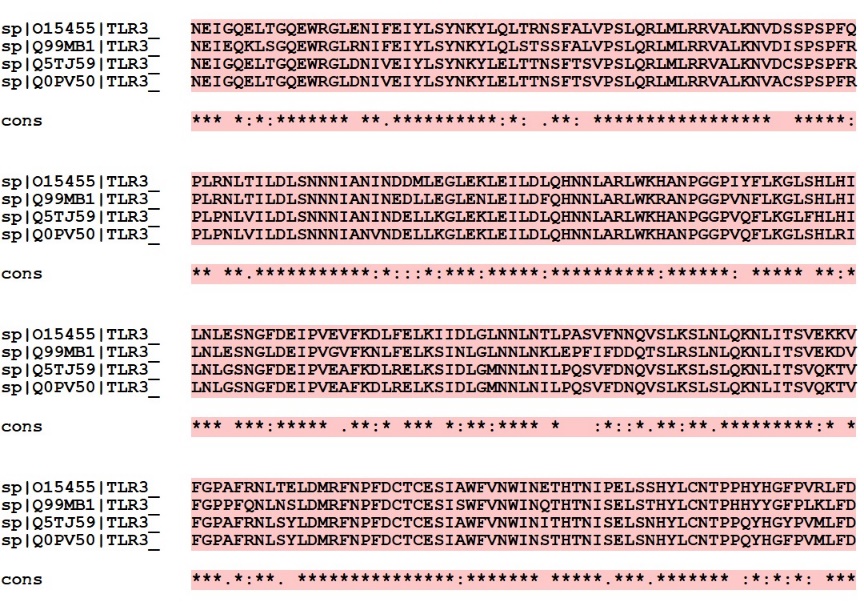


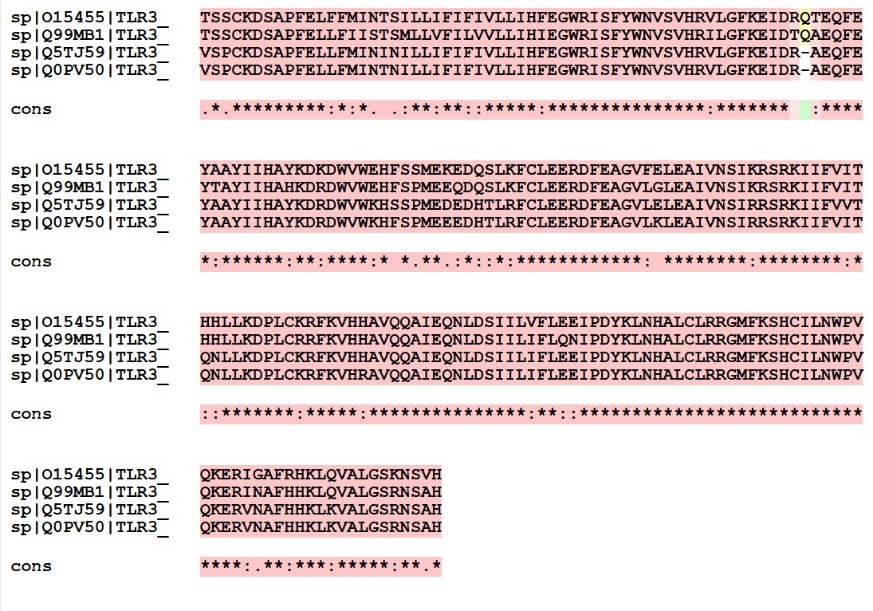


Figure S1: Multiple sequences alignment results using T-Coffee for TLR3 sequence of different species like Homo sapiens, Mus musculus, Bos taurus and Boselaphus tragocamelus with uniport ID O15455, Q99MB1, Q5TJ59 and Q0PV50.

CLUSTER OMEGA

CLUSTAL O(1.2.4) multiple sequence alignment

sp|Q99MB1|TLR3_MOUSE MKGCSSYLMYSFGGLLSLWILLVSSTNQCTVRYNVADCSHLKLTHIPDDLPSNITVLNLT 60

sp|O15455|TLR3_HUMAN MRQTLP-CIYFWGGLLPFGMLCASSTTKCTVSHEVADCSHLKLTQVPDDLPTNITVLNLT 59

sp|Q5TJ59|TLR3_BOVIN MSRPLPYHIHFFSGLLTCWILCTSSAHKCTVRHEVADCSHLKLTQIPDDLPTNITVLNLT 60

sp|Q0PV50|TLR3_BOSTR MSRPLPYHIYFFTGLLTCWILCTSSAHKCTVRHEVADCSHLKLTQIPEDLPTNITVLNLT 60

* :: : *** :* .**: :*** ::**********::*:***:********

sp|Q99MB1|TLR3_MOUSE HNQLRRLPPTNFTRYSQLAILDAGFNSISKLEPELCQILPLLKVLNLQHNELSQISDQTF 120

sp|O15455|TLR3_HUMAN HNQLRRLPAANFTRYSQLTSLDVGFNTISKLEPELCQKLPMLKVLNLQHNELSQLSDKTF 119

sp|Q5TJ59|TLR3_BOVIN HNQLRRLPPANFTRYSQLTTLDGGFNSISKLEPELCQSLPWLEILNLQHNEISQLSDKTF 120

sp|Q0PV50|TLR3_BOSTR HNQLRRLPPANFTRYSRLTILDGGFNSISKLEPELCQNLPWLEILNLQHNEISQLSDKTF 120

******** :******:*: ** ***:********** ** *::*******:**:**:**

sp|Q99MB1|TLR3_MOUSE VFCTNLTELDLMSNSIHKIKSNPFKNQKNLIKLDLSHNGLSSTKLGTGVQLENLQELLLA 180

sp|O15455|TLR3_HUMAN AFCTNLTELHLMSNSIQKIKNNPFVKQKNLITLDLSHNGLSSTKLGTQVQLENLQELLLS 179

sp|Q5TJ59|TLR3_BOVIN IFCMNLTELHLMSNSIQKIKNDPFKNLKNLIKLDLSHNGLSSTKLGTQLQLENLQELLLS 180

sp|Q0PV50|TLR3_BOSTR VFCMNLTELHLMSNSIQKIQNDPFKNLKNLIKLDLSHNGLSSTKLGTQLQLENLQELLLS 180

** *****.******:**:.:** : ****.*************** :**********:

sp|Q99MB1|TLR3_MOUSE KNKILALRSEELEFLGNSSLRKLDLSSNPLKEFSPGCFQTIGKLFALLLNNAQLNPHLTE 240

sp|O15455|TLR3_HUMAN NNKIQALKSEELDIFANSSLKKLELSSNQIKEFSPGCFHAIGRLFGLFLNNVQLGPSLTE 239

sp|Q5TJ59|TLR3_BOVIN NNKISSLTPGEFDFLGNSSLKRLELSSNQIKEFSPGCFHTLGELSGLSLNNAKLSPSLTE 240

sp|Q0PV50|TLR3_BOSTR NNKISSLTPEELDFLGNSSLERLELSSNQIKEFSPGCFHAIGKLSGLSLNNAKLSPSLTE 240

:*** :* *::::.****.:*:**** :********:::*.* .* ***.:*.* ***

sp|Q99MB1|TLR3_MOUSE KLCWELSNTSIQNLSLANNQLLATSESTFSGLKWTNLTQLDLSYNNLHDVGNGSFSYLPS 300

sp|O15455|TLR3_HUMAN KLCLELANTSIRNLSLSNSQLSTTSNTTFLGLKWTNLTMLDLSYNNLNVVGNDSFAWLPQ 299

sp|Q5TJ59|TLR3_BOVIN KLCLELSNTSIENLSLSSNQLDTISHTTFDGLKQTNLTTLDLSRNSLRVMGNDSFAWLPH 300

sp|Q0PV50|TLR3_BOSTR KLCLELSNTSIENLSLSSNQLDTISHTTFSGLKQTNLTTLDLSRSSLRVMDNDSFAWLPH 300

*** **:****.****:..** : *.:** *** **** **** ..*. :.*.**::**

sp|Q99MB1|TLR3_MOUSE LRYLSLEYNNIQRLSPRSFYGLSNLRYLSLKRAFTKQSVSLASHPNIDDFSFQWLKYLEY 360

sp|O15455|TLR3_HUMAN LEYFFLEYNNIQHLFSHSLHGLFNVRYLNLKRSFTKQSISLASLPKIDDFSFQWLKCLEH 359

sp|Q5TJ59|TLR3_BOVIN LEYLSLEYNNIEHLSSRSFYGLSNLRRLDLRRSFTRQSISLTSLPKIDDFSFQWLKCLEY 360

sp|Q0PV50|TLR3_BOSTR LEYLSLEYNNIEHLSSRSFYGLSNLRHLNLRWSFTRQSISLTSLPKIDDFSFQWLKCLEY 360

*.*: ******::* :*::** *:* *.*: :**:**:**:* *:********** **:

sp|Q99MB1|TLR3_MOUSE LNMDDNNIPSTKSNTFTGLVSLKYLSLSKTFTSLQTLTNETFVSLAHSPLLTLNLTKNHI 420

sp|O15455|TLR3_HUMAN LNMEDNDIPGIKSNMFTGLINLKYLSLSNSFTSLRTLTNETFVSLAHSPLHILNLTKNKI 419

sp|Q5TJ59|TLR3_BOVIN LNMDDNNFPGIKRNTFTGLVRLKFLSLSNSFSSLRTLTNETFLSLAGCPLLLLDLTKNKI 420

sp|Q0PV50|TLR3_BOSTR LNMEDNNFPSIKRNTFTGLVRLKFLSLSNSFSSLRTLTNETFLSLAGSPLLLLNLTKNKI 420

***:**::*. * * ****: **:****::*:**:*******:*** .** *:****:*

sp|Q99MB1|TLR3_MOUSE SKIANGTFSWLGQLRILDLGLNEIEQKLSGQEWRGLRNIFEIYLSYNKYLQLSTSSFALV 480

sp|O15455|TLR3_HUMAN SKIESDAFSWLGHLEVLDLGLNEIGQELTGQEWRGLENIFEIYLSYNKYLQLTRNSFALV 479

sp|Q5TJ59|TLR3_BOVIN SKIQSGAFSWLGHLEVLDLGLNEIGQELTGQEWRGLDNIVEIYLSYNKYLELTTNSFTSV 480

sp|Q0PV50|TLR3_BOSTR SKIQSGAFSWLGHLEVLDLGLNEIGQELTGQEWRGLDNIVEIYLSYNKYLELTTNSFTSV 480

*** ..:*****:*.:******** *:*:******* **.**********:*: .**: *

sp|Q99MB1|TLR3_MOUSE PSLQRLMLRRVALKNVDISPSPFRPLRNLTILDLSNNNIANINEDLLEGLENLEILDFQH 540

sp|O15455|TLR3_HUMAN PSLQRLMLRRVALKNVDSSPSPFQPLRNLTILDLSNNNIANINDDMLEGLEKLEILDLQH 539

sp|Q5TJ59|TLR3_BOVIN PSLQRLMLRRVALKNVDCSPSPFRPLPNLVILDLSNNNIANINDELLKGLEKLEILDLQH 540

sp|Q0PV50|TLR3_BOSTR PSLQRLMLRRVALKNVACSPSPFRPLPNLVILDLSNNNIANVNDELLKGLEKLEILDLQH 540

**************** *****:** **.***********:*:::*:***:*****:**

sp|Q99MB1|TLR3_MOUSE NNLARLWKRANPGGPVNFLKGLSHLHILNLESNGLDEIPVGVFKNLFELKSINLGLNNLN 600

sp|O15455|TLR3_HUMAN NNLARLWKHANPGGPIYFLKGLSHLHILNLESNGFDEIPVEVFKDLFELKIIDLGLNNLN 599

sp|Q5TJ59|TLR3_BOVIN NNLARLWKHANPGGPVQFLKGLFHLHILNLGSNGFDEIPVEAFKDLRELKSIDLGMNNLN 600

sp|Q0PV50|TLR3_BOSTR NNLARLWKHANPGGPVQFLKGLSHLRILNLGSNGFDEIPVEAFKDLRELKSIDLGMNNLN 600

********:******: ***** **:**** ***:***** .**:* *** *:**:****

sp|Q99MB1|TLR3_MOUSE KLEPFIFDDQTSLRSLNLQKNLITSVEKDVFGPPFQNLNSLDMRFNPFDCTCESISWFVN 660

sp|O15455|TLR3_HUMAN TLPASVFNNQVSLKSLNLQKNLITSVEKKVFGPAFRNLTELDMRFNPFDCTCESIAWFVN 659

sp|Q5TJ59|TLR3_BOVIN ILPQSVFDNQVSLKSLSLQKNLITSVQKTVFGPAFRNLSYLDMRFNPFDCTCESIAWFVN 660

sp|Q0PV50|TLR3_BOSTR ILPQSVFDNQVSLKSLSLQKNLITSVQKTVFGPAFRNLSYLDMRFNPFDCTCESIAWFVN 660

* :*::*.**:**.*********:* **** *:**. ***************:****

sp|Q99MB1|TLR3_MOUSE WINQTHTNISELSTHYLCNTPHHYYGFPLKLFDTSSCKDSAPFELLFIISTSMLLVFILV 720

sp|O15455|TLR3_HUMAN WINETHTNIPELSSHYLCNTPPHYHGFPVRLFDTSSCKDSAPFELFFMINTSILLIFIFI 719

sp|Q5TJ59|TLR3_BOVIN WINITHTNISELSNHYLCNTPPQYHGYPVMLFDVSPCKDSAPFELLFMININILLIFIFI 720

sp|Q0PV50|TLR3_BOSTR WINSTHTNISELSNHYLCNTPPQYHGFPVMLFDVSPCKDSAPFELLFMINTNILLIFIFI 720

*** ***** ***.******* :*:*:*: ***.* *********:*:*. .:**:**::

sp|Q99MB1|TLR3_MOUSE VLLIHIEGWRISFYWNVSVHRILGFKEIDTQAEQFEYTAYIIHAHKDRDWVWEHFSPMEE 780

sp|O15455|TLR3_HUMAN VLLIHFEGWRISFYWNVSVHRVLGFKEIDRQTEQFEYAAYIIHAYKDKDWVWEHFSSMEK 779

sp|Q5TJ59|TLR3_BOVIN VLLIHFEGWRISFYWNVSVHRVLGFKEIDR-AEQFEYAAYIIHAYKDRDWVWKHSSPMED 779

sp|Q0PV50|TLR3_BOSTR VLLIHFEGWRISFYWNVSVHRVLGFKEIDR-AEQFEYAAYIIHAYKDRDWVWKHFSPMEE 779

*****:***************:******* :*****:******:**:****:* * **.

sp|Q99MB1|TLR3_MOUSE QDQSLKFCLEERDFEAGVLGLEAIVNSIKRSRKIIFVITHHLLKDPLCRRFKVHHAVQQA 840

sp|O15455|TLR3_HUMAN EDQSLKFCLEERDFEAGVFELEAIVNSIKRSRKIIFVITHHLLKDPLCKRFKVHHAVQQA 839

sp|Q5TJ59|TLR3_BOVIN EDHTLRFCLEERDFEAGVLELEAIVNSIRRSRKIIFVVTQNLLKDPLCKRFKVHHAVQQA 839

sp|Q0PV50|TLR3_BOSTR EDHTLRFCLEERDFEAGVLKLEAIVNSIRRSRKIIFVITQNLLKDPLCKRFKVHRAVQQA 839

:*::*:************: ********:********:*::*******:*****:*****

sp|Q99MB1|TLR3_MOUSE IEQNLDSIILIFLQNIPDYKLNHALCLRRGMFKSHCILNWPVQKERINAFHHKLQVALGS 900

sp|O15455|TLR3_HUMAN IEQNLDSIILVFLEEIPDYKLNHALCLRRGMFKSHCILNWPVQKERIGAFRHKLQVALGS 899

sp|Q5TJ59|TLR3_BOVIN IEQNLDSIILIFLEEIPDYKLNHALCLRRGMFKSHCILNWPVQKERVNAFHHKLKVALGS 899

sp|Q0PV50|TLR3_BOSTR IEQNLDSIILIFLEEIPDYKLNHALCLRRGMFKSHCILNWPVQKERVNAFHHKLKVALGS 899

**********:**::*******************************:.**:***:*****

sp|Q99MB1|TLR3_MOUSE RNSAH 905

sp|O15455|TLR3_HUMAN KNSVH 904

sp|Q5TJ59|TLR3_BOVIN RNSAH 904

sp|Q0PV50|TLR3_BOSTR RNSAH 904

:**.*

Figure S2: Multiple sequences alignment results using CLUSTAL-OMEGAfor TLR3 sequence of different species like Homo sapiens, Mus musculus, Bos taurus and Boselaphus tragocamelus with uniport ID O15455, Q99MB1, Q5TJ59 and Q0PV50

NETSURF RESULTS

1. N284I


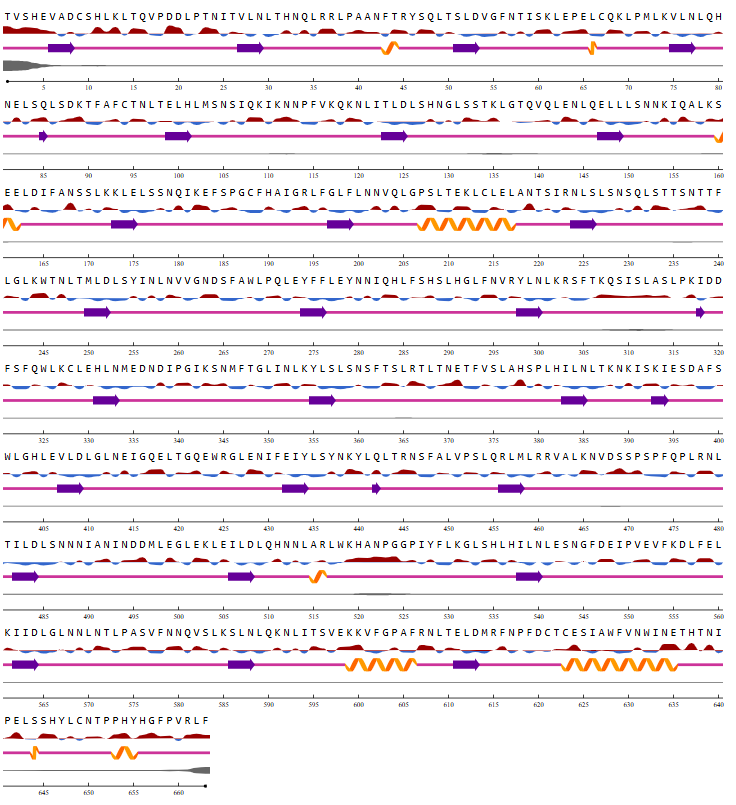


1. C37R


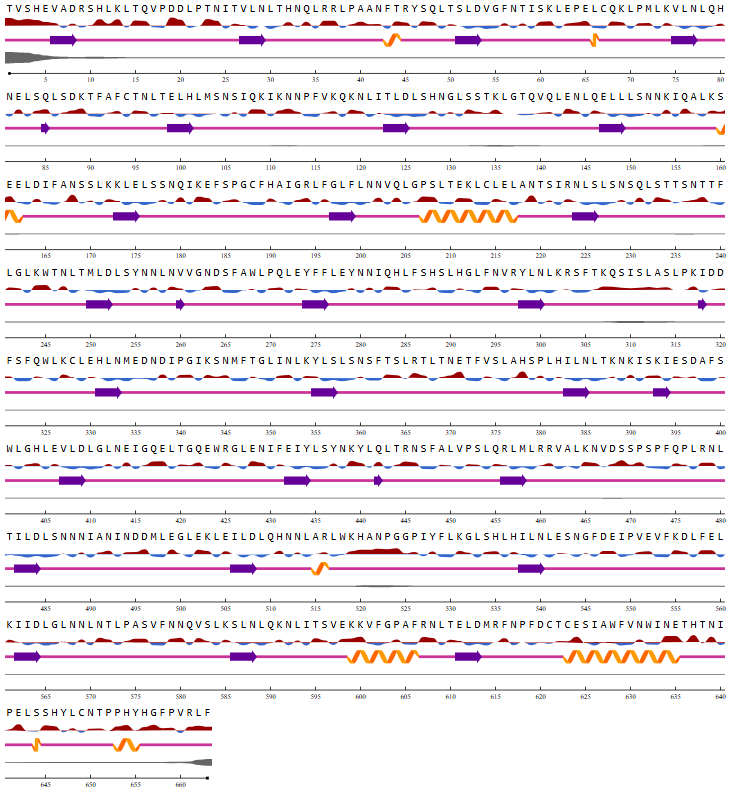


1. L360P


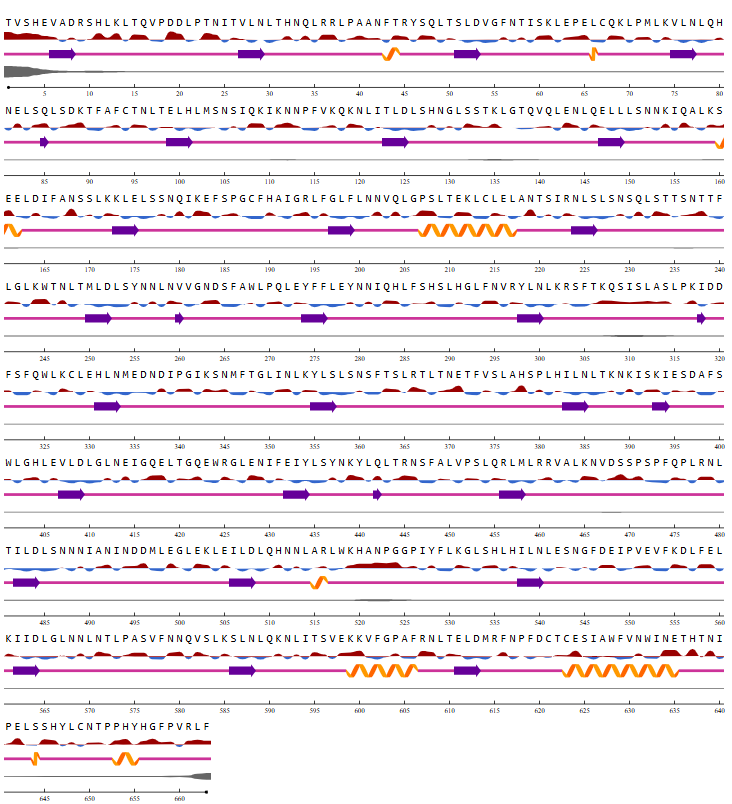


1. Q538I


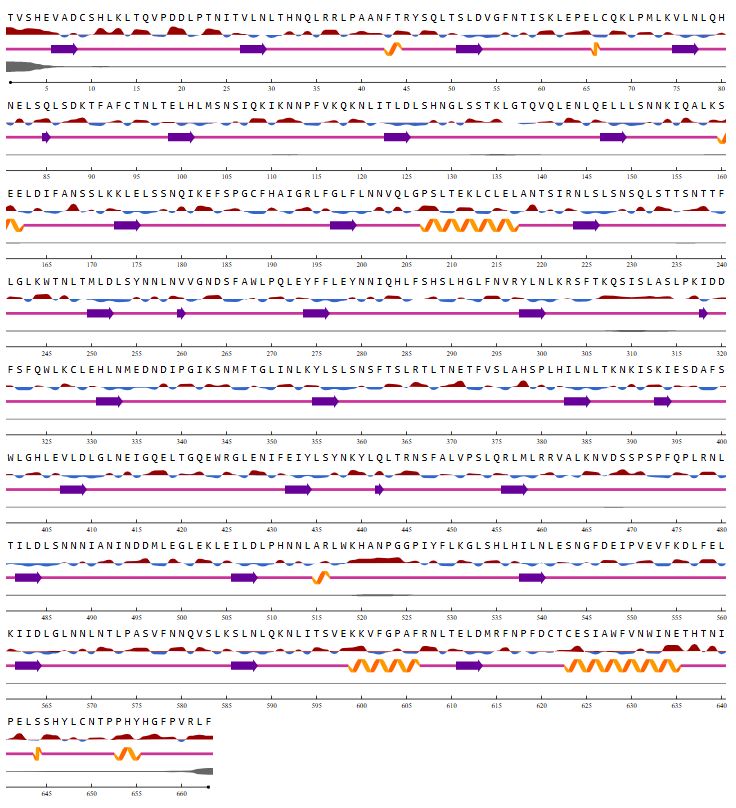


Figure S3: Net Surf result analysis of selected SNPs a) N284I, b) C37R, c) L360P, and d) Q538P.

Table S1: FATHMM-MKL based analysis of selected 4 mutations

| Chromoso4me | Mutation | Position | Variant | Non-Coding Score | Non-Coding Groups | Coding Score | Coding Groups | Further Information |
| --- | --- | --- | --- | --- | --- | --- | --- | --- |
| 4 | N284I | 18703691 | A/T | 0.99253 | ADB | 0.99256 | AEFDBI | Highly likely pathogenic |
| 4 | C37R | 186997882 | T/C | 0.99346 | AB | 0.98728 | AEFBI | Strong evidence of being deleterious, especially with high non-coding impact |
| 4 | Q538P | 18704453 | A/C | 0.99086 | AB | 0.98311 | AEFBI | High likelihood of functional disruption |
| 4 | L360P | 187003919 | T/C | 0.99325 | AB | 0.99103 | AEFBI | significant pathogenic potential in both coding and non-coding |

Predictions are given as p-values in the range [0, 1]: values above 0.5 are predicted to be deleterious, while those below 0.5 are predicted to be neutral or benign. P-values close to the extremes (0 or 1) are the highest-confidence predictions that yield the highest accuracy.

Table S2: FATHMM-XF based analysis of selected 4 mutation

| Mutation | \| Chromosome \|  \|  \| \| --- \| --- \| --- \| | Position | Variant | \|  \|  \| Coding Score \| \| --- \| --- \| --- \| | Further Information |
| --- | --- | --- | --- | --- | --- | --- | --- | --- | --- | --- | --- |
| N284I | 4 | 187003691 | A/T | 0.871397 | \| pathogenic \| \| --- \| |
| C37R | 4 | 186997882 | \| T/C \|  \| \| --- \| --- \| | 0.937443 | \| pathogenic \| \| --- \| |
| Q538P | 4 | 187004453 | A/C | - | \|  \| No prediction found \| \| --- \| --- \| |
| L360P | 4 | 187003919 | T/C | \| 0.934636 \| \| --- \| | \| pathogenic \| \| --- \| |

Predictions are given as p-values in the range [0, 1]: values above 0.5 are predicted to be deleterious, while those below 0.5 are predicted to be neutral or benign. P-values close to the extremes (0 or 1) are the highest-confidence predictions that yield the highest accuracy.

RAMAPLOT DIFFERENT RESULTS

5GS0


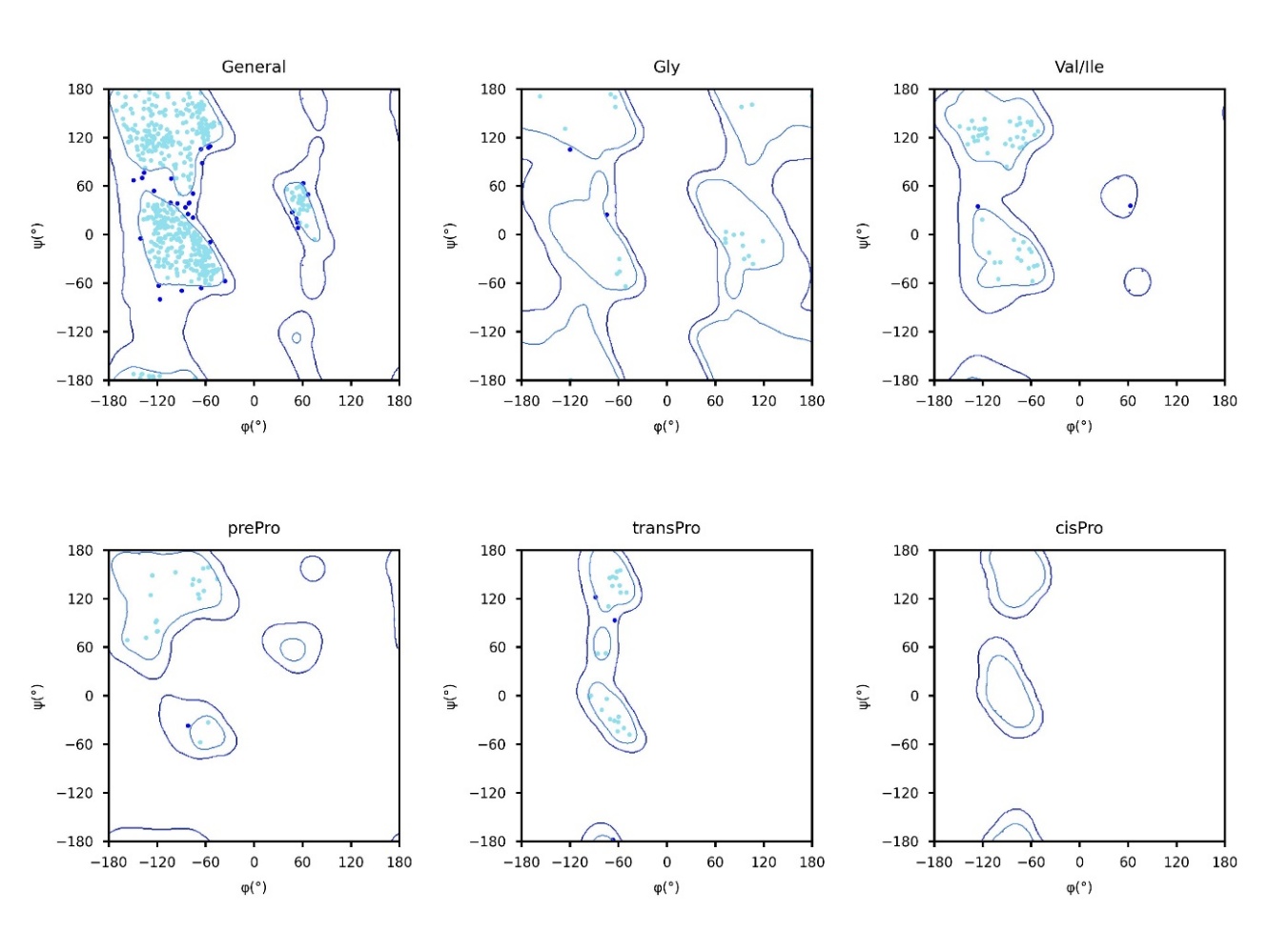


Figure S4.1: 2D Ramachandran plot of six distinct categories of 5GS0: (a) general case (Ala and remaining 15 amino acids), (b) Gly, (c) Val/Ile, (d) pre-Pro, (e) trans-Pro & (f) cis-Pro.
Here, cyan blue and red dots represent torsion angles of favoured, allowed and disallowed regions respectively.


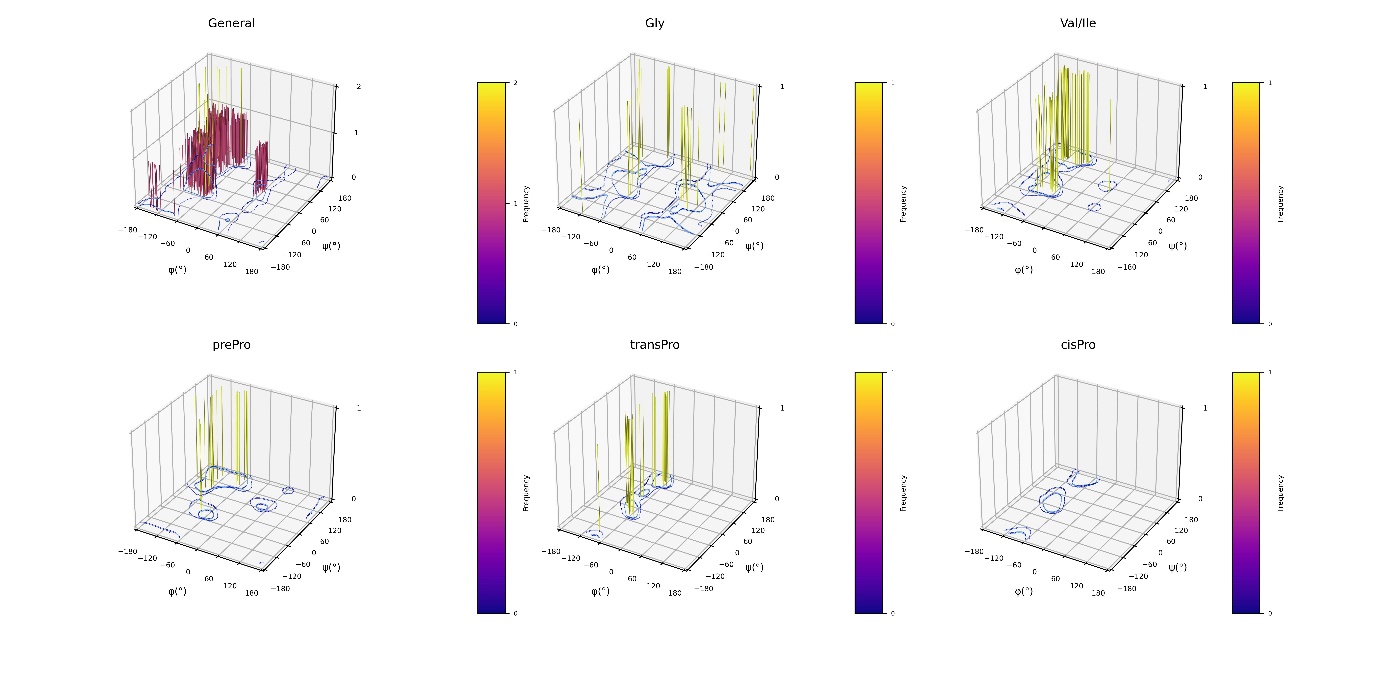
Figure S4.2: 3D Ramachandran plot of six distinct categories of 5GS0: (a) general case (Ala and remaining 15 amino acids), (b) Gly, (c) Val/Ile, (d) pre-Pro, (e) trans-Pro & (f) cis-Pro.
Here, bars represent frequency of torsion angles.

L360P


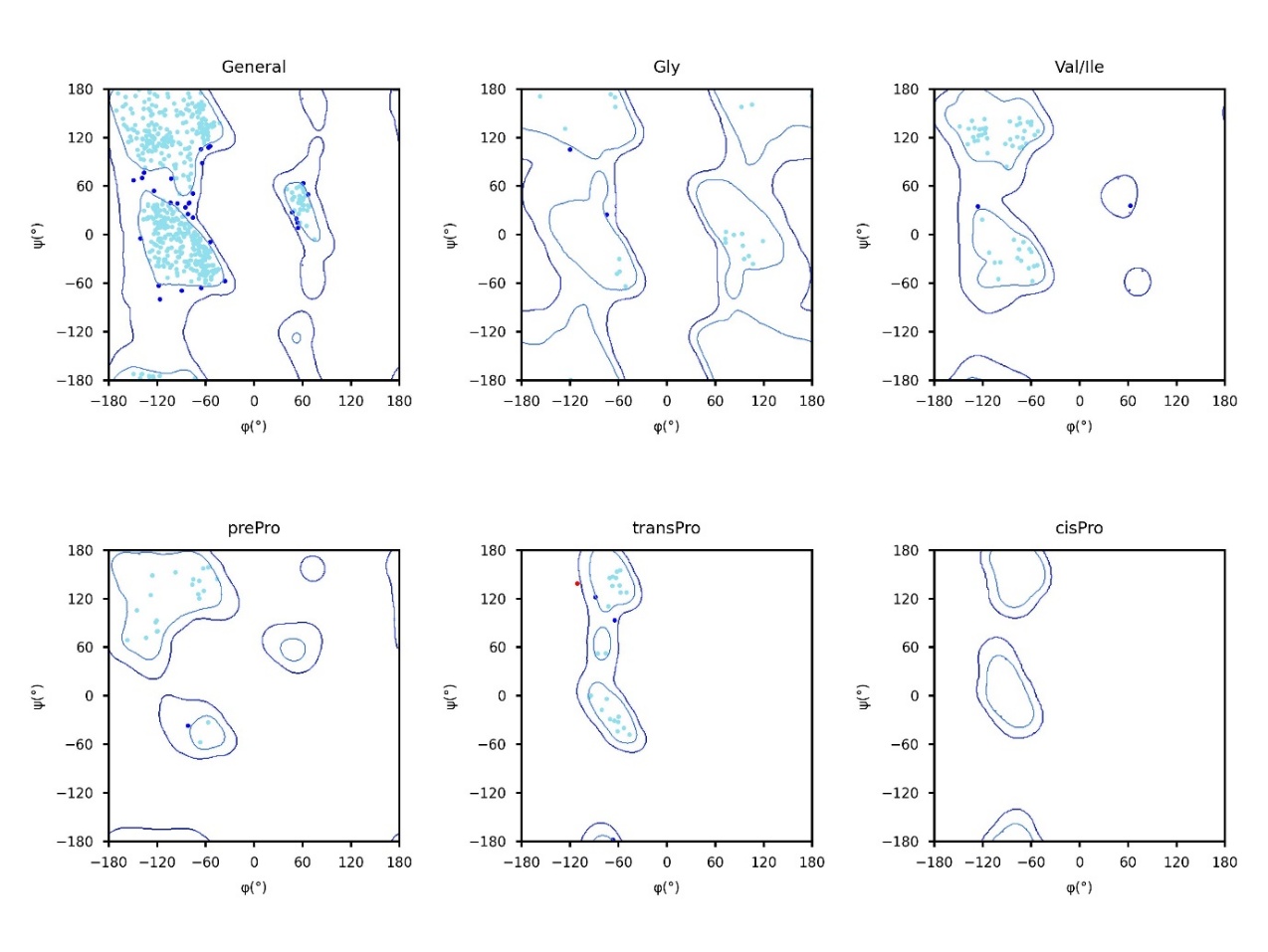


Figure S5.1: 2D Ramachandran plot of six distinct categories of L360P: (a) general case (Ala and remaining 15 amino acids), (b) Gly, (c) Val/Ile, (d) pre-Pro, (e) trans-Pro & (f) cis-Pro.
Here, cyan blue and red dots represent torsion angles of favoured, allowed and disallowed regions respectively.


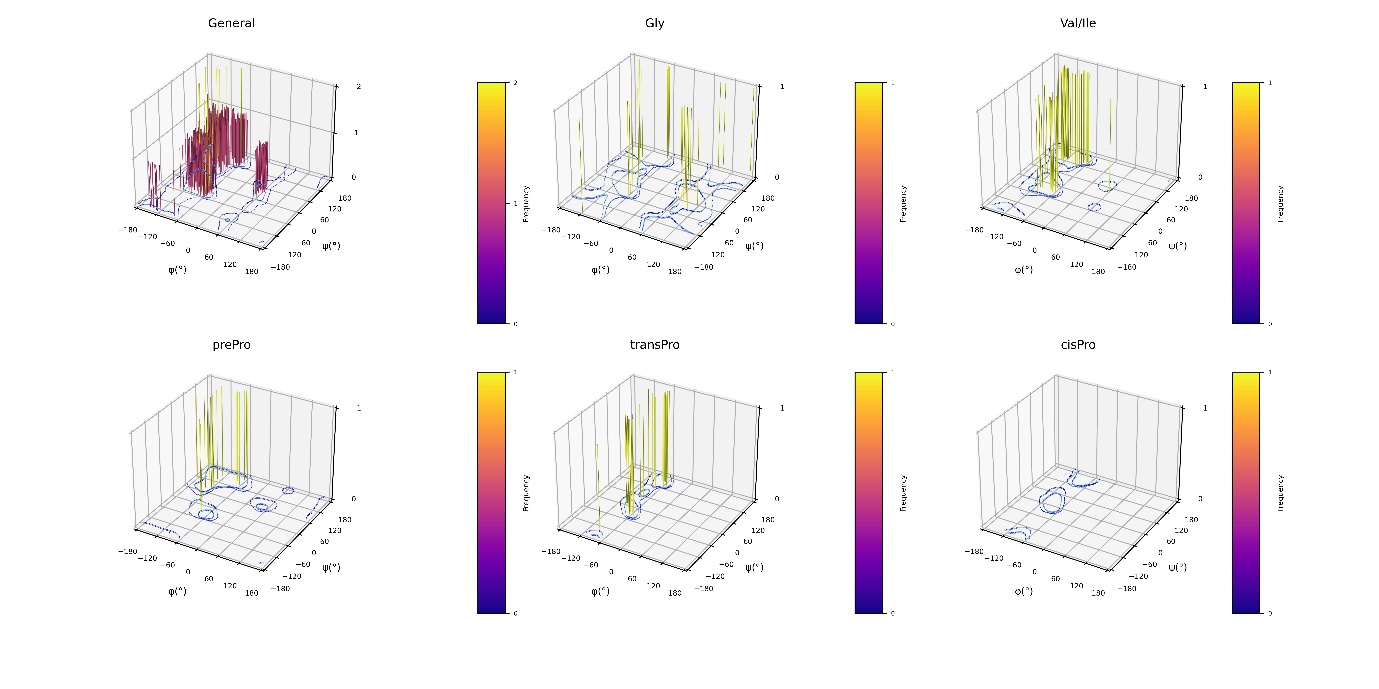


Figure S5.2: 3D Ramachandran plot of six distinct categories of L360P: (a) general case (Ala and remaining 15 amino acids), (b) Gly, (c) Val/Ile, (d) pre-Pro, (e) trans-Pro & (f) cis-Pro.
Here, bars represent frequency of torsion angles.

N248I


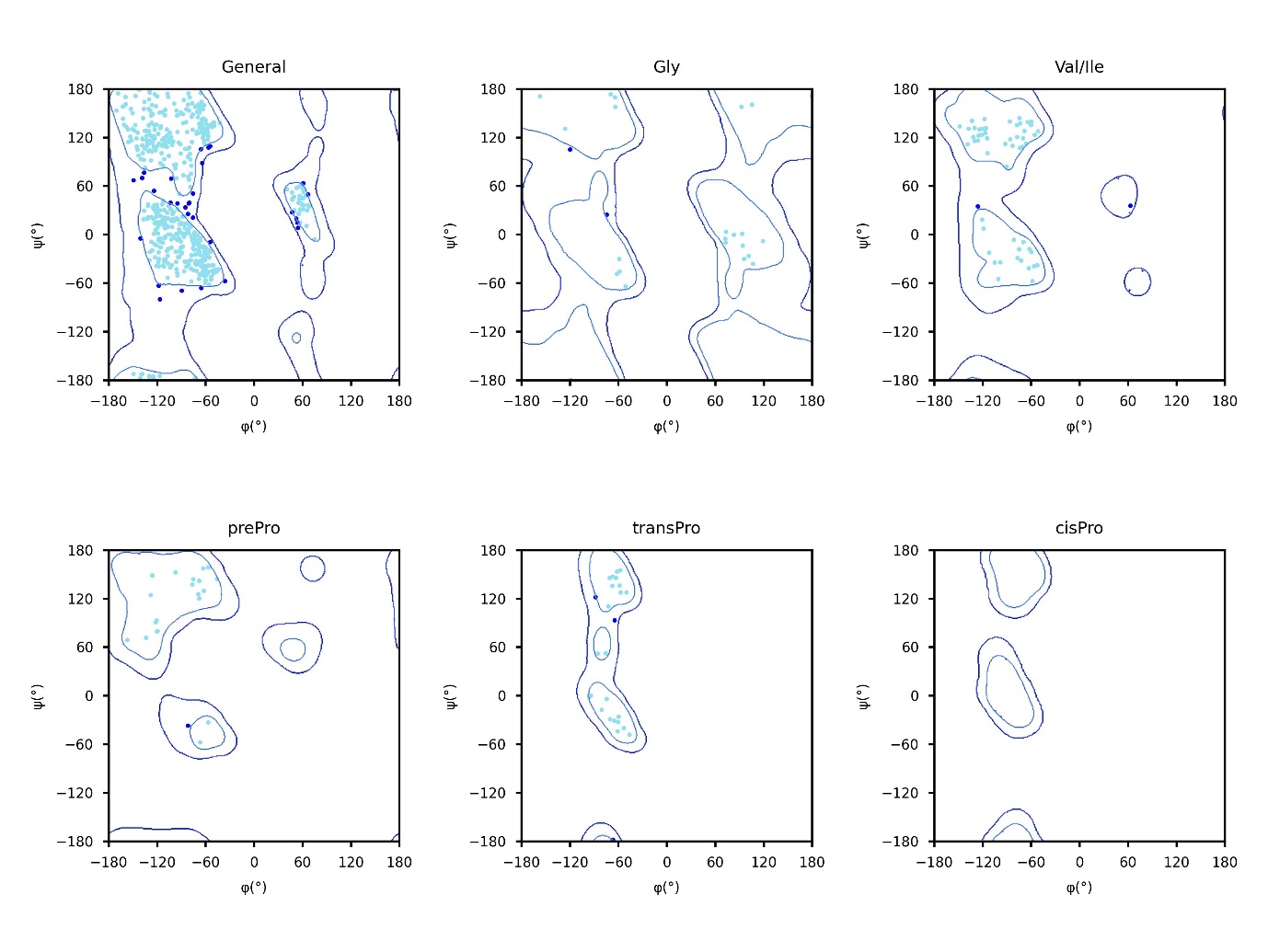


Figure S6.1: 2D Ramachandran plot of six distinct categories of N248I: (a) general case (Ala and remaining 15 amino acids), (b) Gly, (c) Val/Ile, (d) pre-Pro, (e) trans-Pro & (f) cis-Pro.
Here, cyan blue and red dots represent torsion angles of favoured, allowed and disallowed regions respectively.


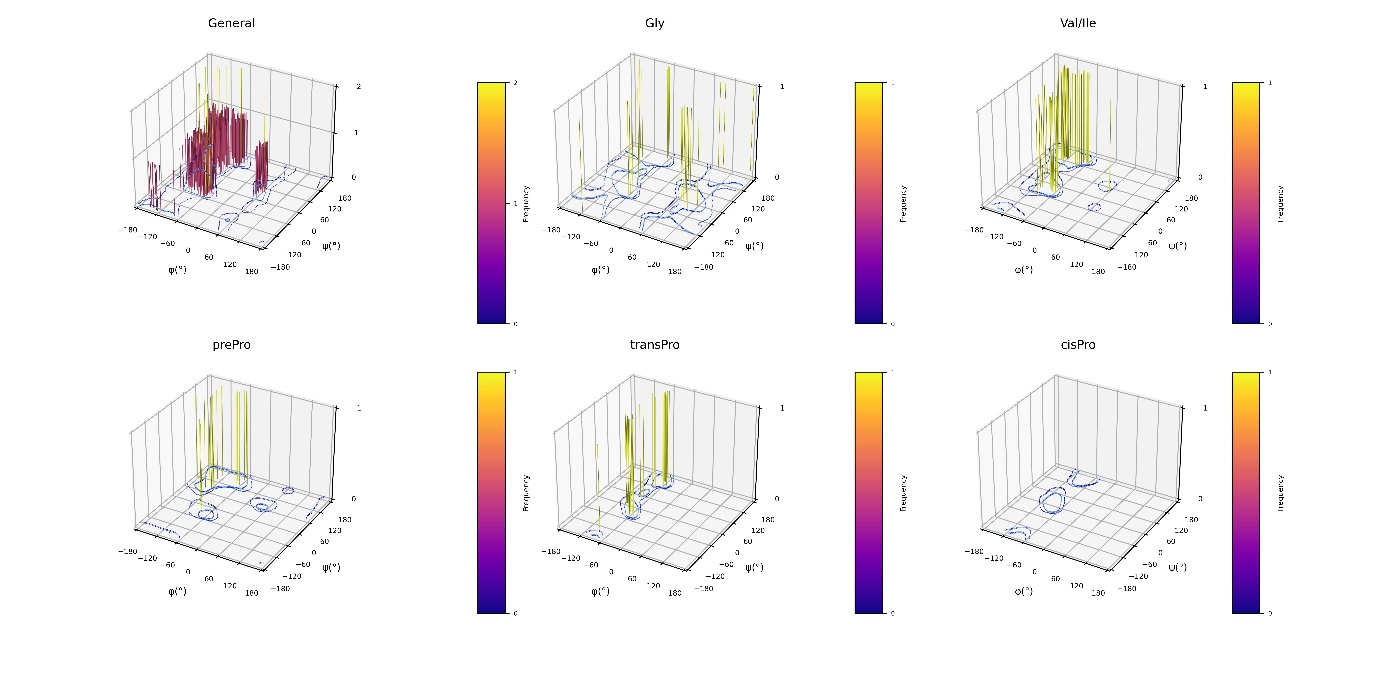


Figure S6.2: 3D Ramachandran plot of six distinct categories of N248I: (a) general case (Ala and remaining 15 amino acids), (b) Gly, (c) Val/Ile, (d) pre-Pro, (e) trans-Pro & (f) cis-Pro.
Here, bars represent frequency of torsion angles.

C37R


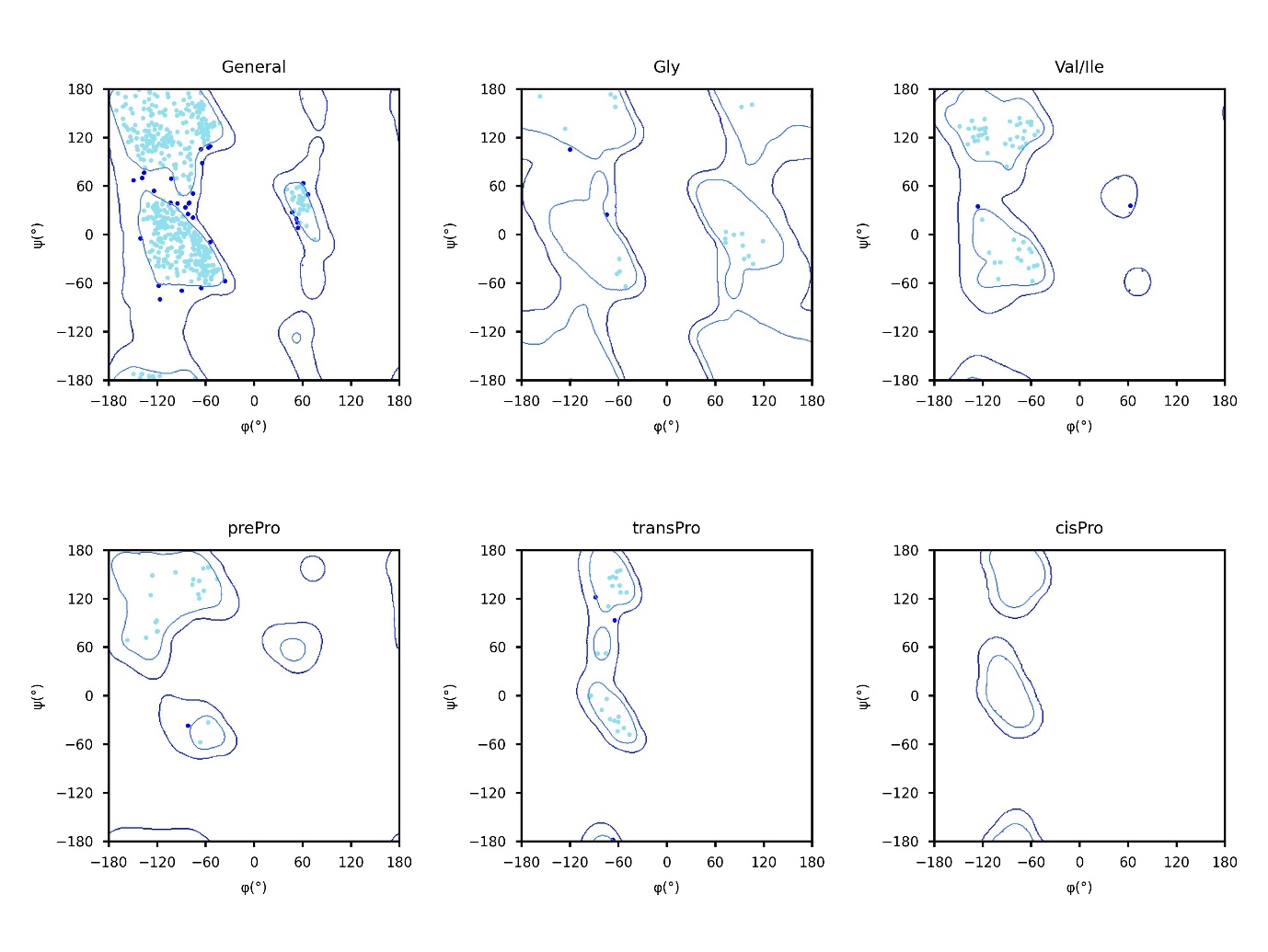


Figure S7.1: 2D Ramachandran plot of six distinct categories of C37R: (a) general case (Ala and remaining 15 amino acids), (b) Gly, (c) Val/Ile, (d) pre-Pro, (e) trans-Pro & (f) cis-Pro.
Here, cyan blue and red dots represent torsion angles of favoured, allowed and disallowed regions respectively.


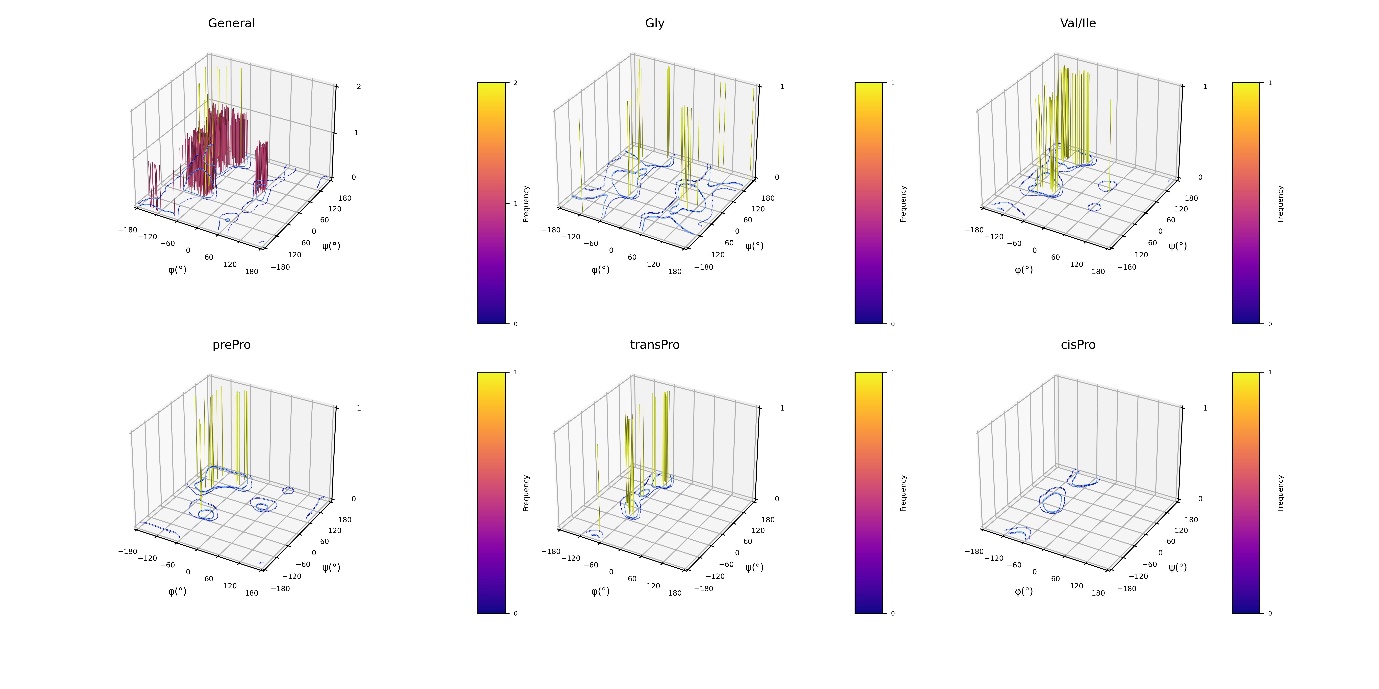


Figure S7.2: 3D Ramachandran plot of six distinct categories of C37R: (a) general case (Ala and remaining 15 amino acids), (b) Gly, (c) Val/Ile, (d) pre-Pro, (e) trans-Pro & (f) cis-Pro.
Here, bars represent frequency of torsion angles.

Q538P


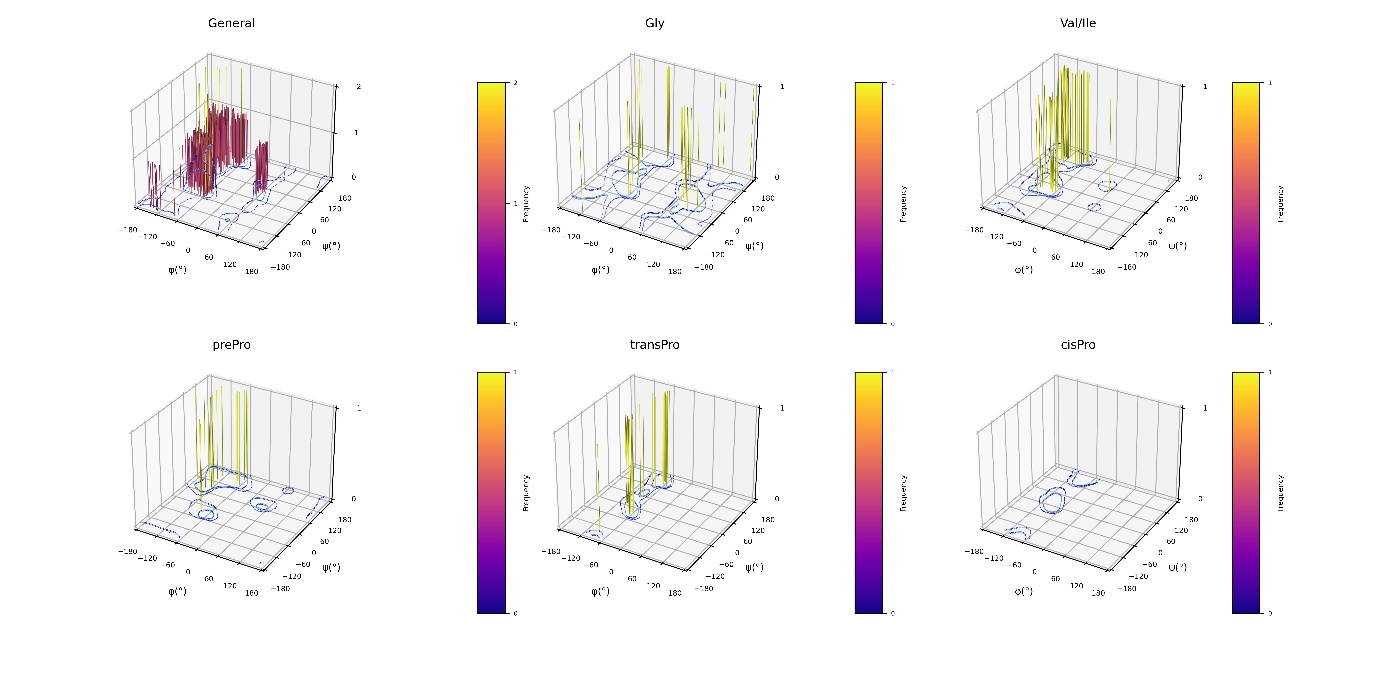


Figure S8.1: 3D Ramachandran plot of six distinct categories of Q538P: (a) general case (Ala and remaining 15 amino acids), (b) Gly, (c) Val/Ile, (d) pre-Pro, (e) trans-Pro & (f) cis-Pro.
Here, bars represent frequency of torsion angles.


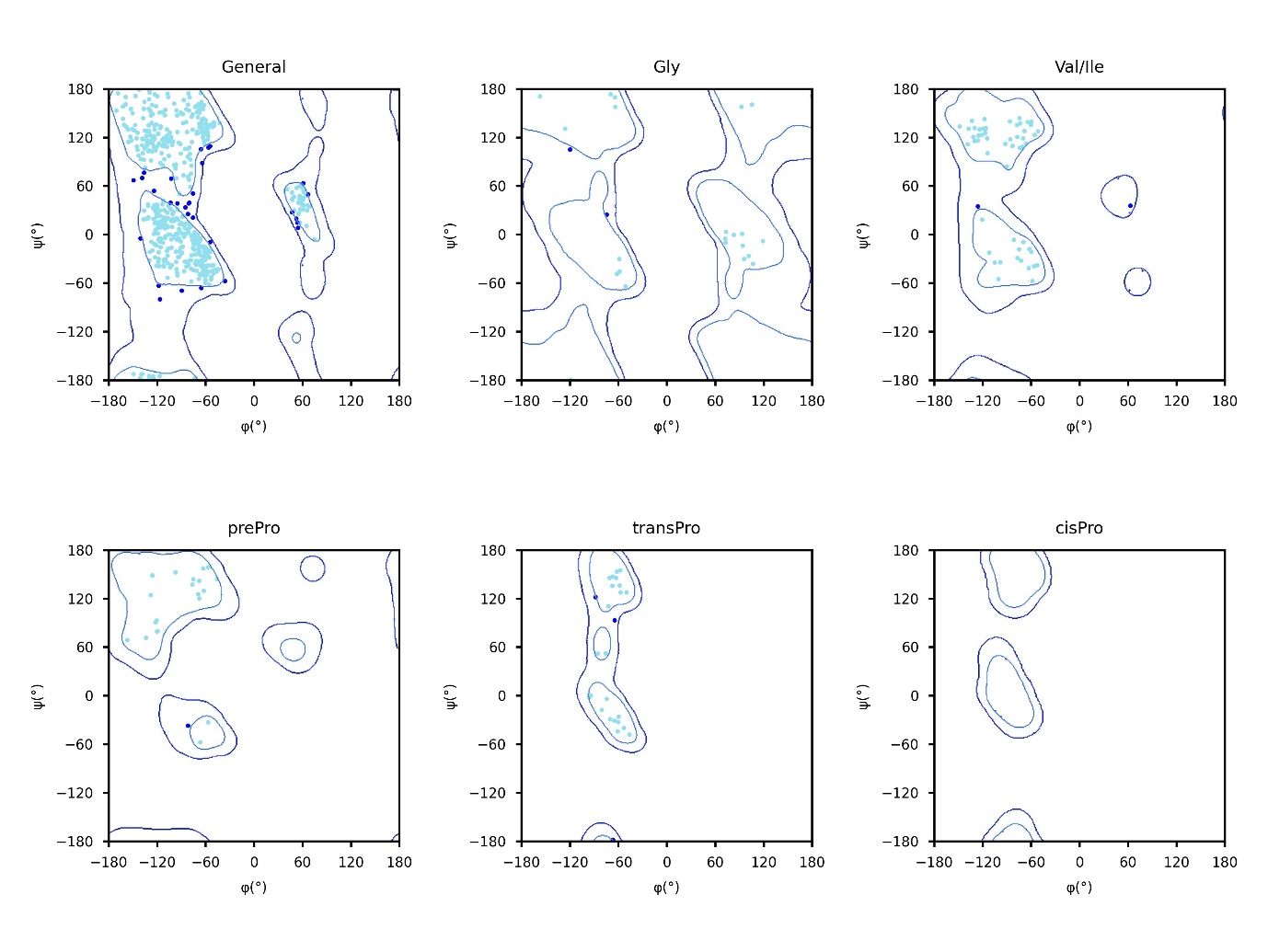


Figure S8.2: 2D Ramachandran plot of six distinct categories of Q538P: (a) general case (Ala and remaining 15 amino acids), (b) Gly, (c) Val/Ile, (d) pre-Pro, (e) trans-Pro & (f) cis-Pro.
Here, cyan blue and red dots represent torsion angles of favoured, allowed and disallowed regions respectively.


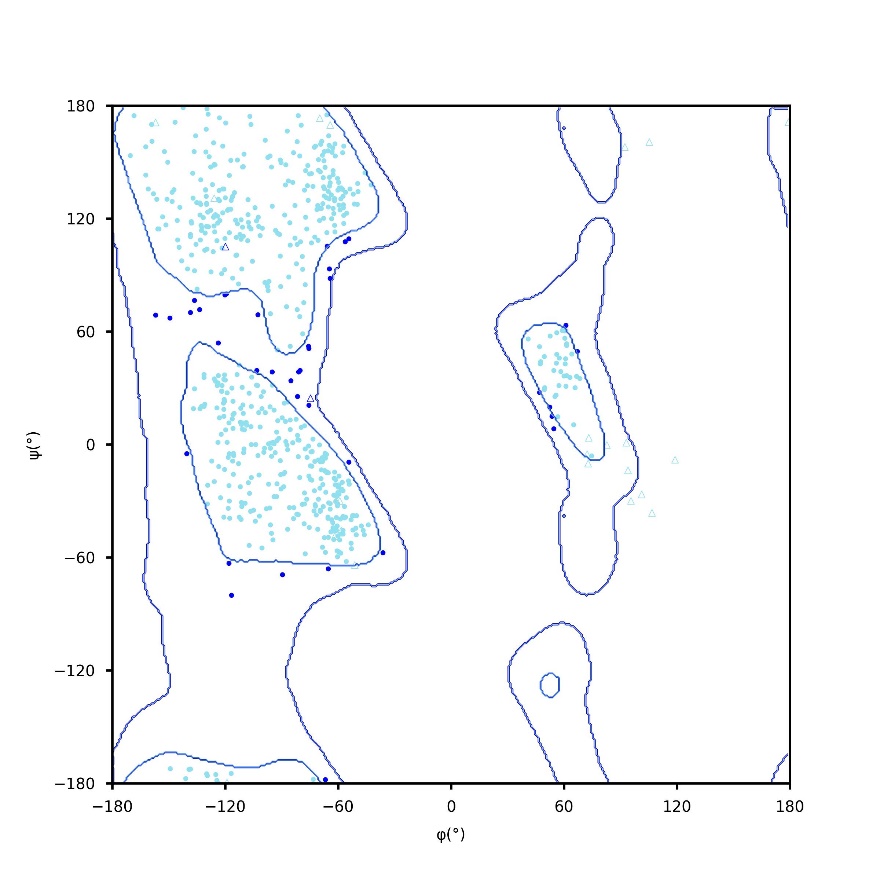


Figure S8.3: Standard 2D Ramachandran Plot of Q538P
Here, cyan, blue and red (dots/triangles) represent torsion angles of favoured, allowed and disallowed regions respectively; dot represents residues other than glycine and triangles represents glycine.


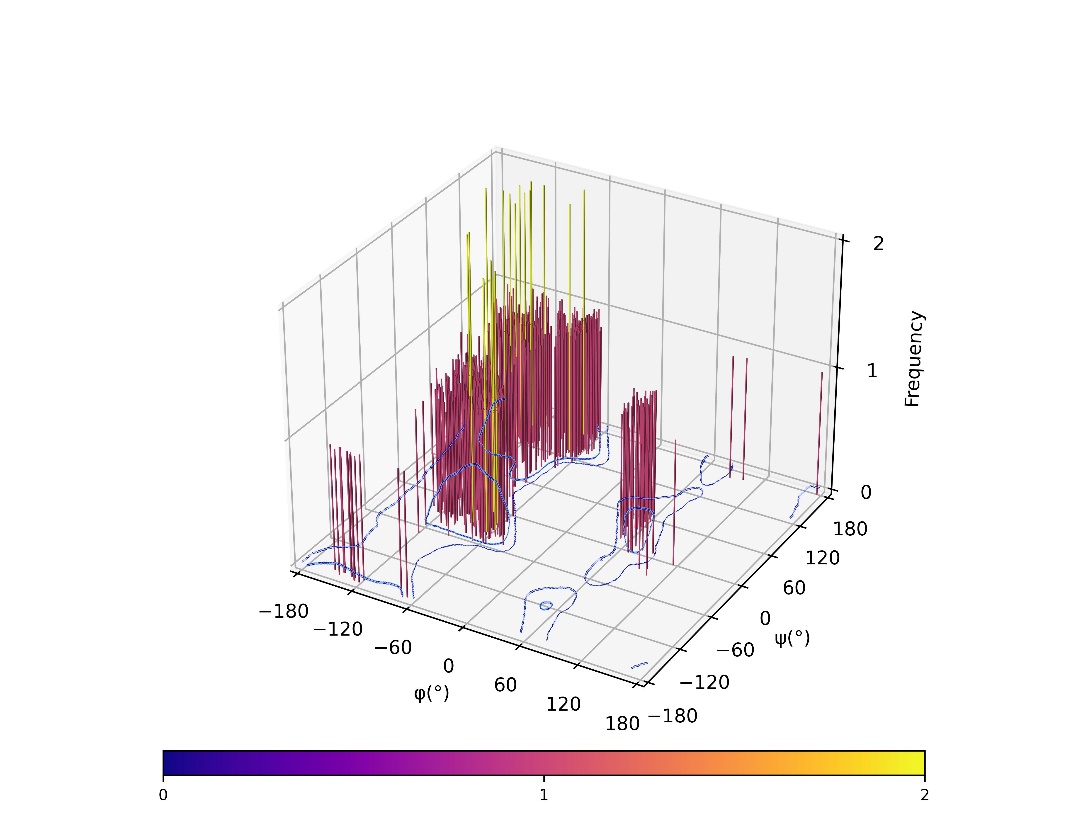


Figure S8.4: Standard 3D Ramachandran plot of Q538P
Here, bar represents frequency of torsion angles.

ALPHAFOLD


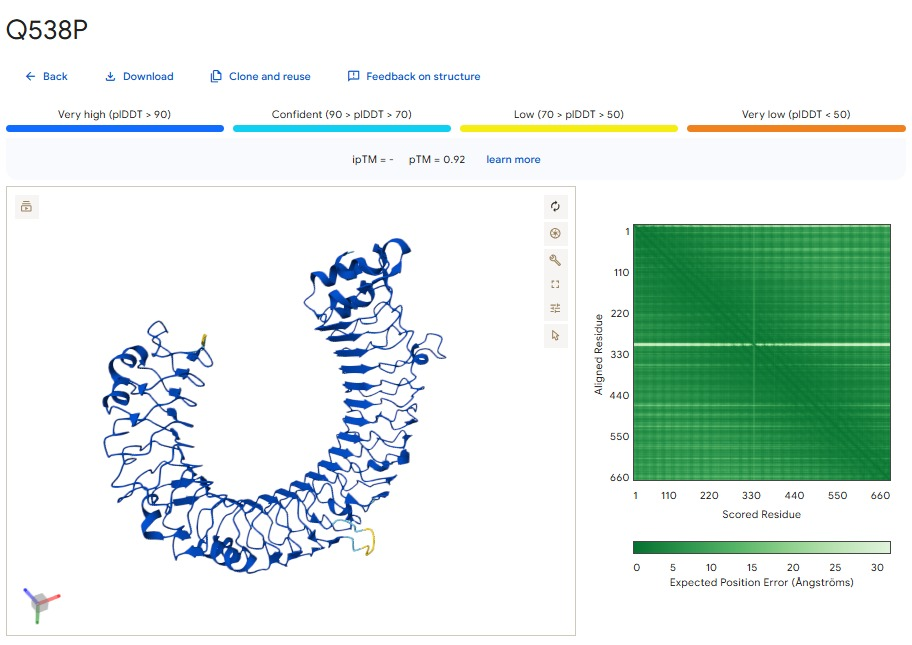


Figure S9: ALPHAFOLD Result of Q538P


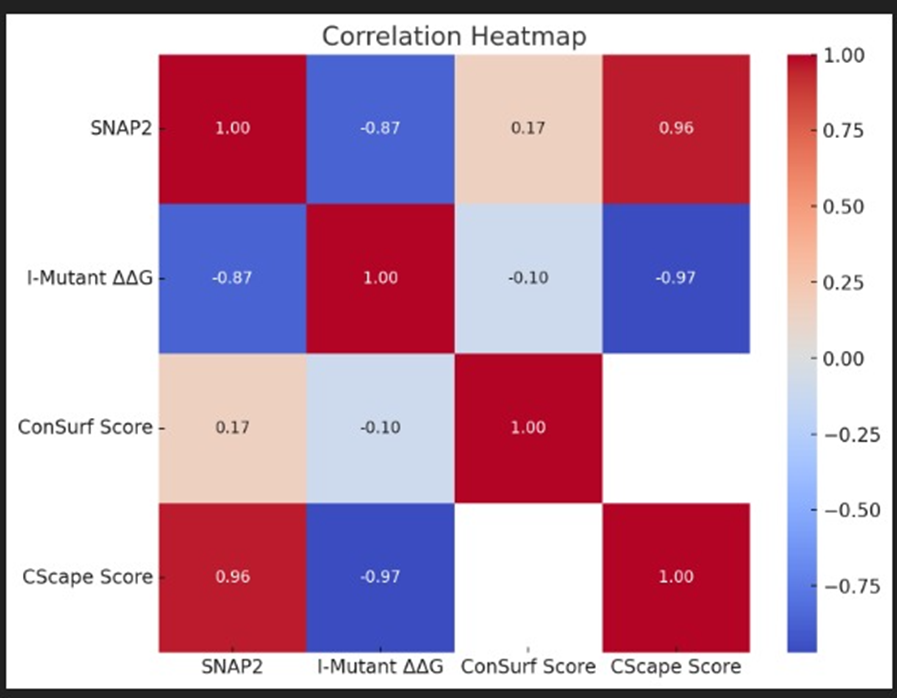


Figure S10: Spearman correlation analysis visualized in the heatmap
